# Health effects of drinking 100% juice: an umbrella review of systematic reviews with meta-analyses

**DOI:** 10.1093/nutrit/nuae036

**Published:** 2024-04-29

**Authors:** Emma L Beckett, Flávia Fayet-Moore, Tim Cassettari, Carlene Starck, Jutta Wright, Michelle Blumfield

**Affiliations:** FOODiQ Global, Sydney, New South Wales, Australia; Food Science & Human Nutrition, School of Environmental and Life Sciences, The University of Newcastle, Central Coast, New South Wales, Australia; FOODiQ Global, Sydney, New South Wales, Australia; Food Science & Human Nutrition, School of Environmental and Life Sciences, The University of Newcastle, Central Coast, New South Wales, Australia; FOODiQ Global, Sydney, New South Wales, Australia; FOODiQ Global, Sydney, New South Wales, Australia; FOODiQ Global, Sydney, New South Wales, Australia; FOODiQ Global, Sydney, New South Wales, Australia

**Keywords:** 100%, fruit juice, chronic disease, dietary guidelines, nutritional epidemiology, systematic review

## Abstract

**Context:**

Low fruit and vegetable intakes are major modifiable determinants of disease. One hundred percent juice may facilitate intake and deliver essential nutrients and bioactive compounds. However, the position of 100% juice in healthy eating guidelines remains controversial due to its lower dietary fiber and higher free-sugar contents compared with whole fruits and vegetables.

**Objective:**

To conduct an umbrella review of systematic literature reviews with meta-analyses (MAs) to summarize the health benefits of drinking 100% fruit and/or vegetable juice.

**Data Sources:**

Four databases (Medline, The Cochrane Library, EMBASE, and CINAHL) were systematically searched for MAs of 100% juice and any health outcomes.

**Data Analysis:**

Screening, quality, risk of bias, and content overlap tools were applied, and extracted data were narratively synthesized. No eligible studies for vegetable juice were found. Fifteen systematic literature reviews (51 primary MAs, 6 dose–response, and 87 subanalyses; 50–1200 mL/day; hours to years of duration) were included. Ten MAs (19.6%) reported health benefits (4 for blood pressure, 2 for vascular function, 3 for inflammation, 1 for stroke mortality), 3 MAs (5.9%) reported adverse risks (1 each for cardiovascular disease mortality, prostate cancer, type 2 diabetes risk), while most (74.5%) reported no effect (blood lipids, body composition, liver function, metabolic health, cancers, and inflammation). Risks were limited to cohort studies and benefits were found in both cohort and intervention studies.

**Conclusion:**

The findings collate evidence showing some potential health benefits associated with 100% juice consumption, with fewer potential risks. The balance of evidence does not support the exclusion of 100% juice from food-based guides to healthy eating, although caution may be warranted in certain groups or individuals, and the body of evidence is not yet conclusive.

**Systematic Review Registration:**

PROSPERO registration no. CRD42022380588.

## INTRODUCTION

Low levels of fruit and vegetable consumption are a major modifiable risk factor for noncommunicable disease,^1^ contributing to approximately 16 million disability-adjusted life-years and millions of deaths worldwide annually.^2^ Globally, the mean intake of fruits and vegetables is approximately 290 g per day, significantly less than the 400 g recommended by the World Health Organization (WHO).^3^ The most recent Australian National Health Survey (2020–2021) revealed that only 6.1% of adults and 8.5% of children consumed the recommended amounts of both fruits and vegetables,^4^ with average daily servings of fruit and vegetables for adults at 1.6 and 2.6, respectively,^4^ compared with the recommended 2 servings of fruit and 5–7.5 servings of vegetables (depending on sex and life stage). As such, diets are potentially lacking in healthful dietary fiber, micronutrients, and bioactive compounds.^5–7^ Barriers to fruit and vegetable consumption include food preferences, habits, access, spoilage, lack of preparation skills, convenience, and cost.^8^^,^^9^ One hundred percent fruit and/or vegetable juices may circumvent these barriers; however, their place in dietary recommendations remains controversial.

While lower in fiber and higher in free sugars than whole fruits and vegetables, 100% juices contain key micronutrients of public health interest, including vitamin C, potassium, and vitamin A,^10^ and important bioactive compounds, such as flavonoids, carotenoids, and other compounds with antioxidant properties.^11–13^ Nutritional properties vary depending on pulp contents, fruit/vegetable variety, ripeness, and processing.^14–16^ Importantly, juicing can contribute to improved access, affordability, and palatability relative to whole fruits and vegetables, with no cooking skills or preparation required, longer shelf-life, and less complex storage and shipping requirements.^12^^,^^13^ Average juice consumption, including 100% juice, in Australia is estimated at 50 mL per capita per day and 275 to 349 mL per capita per day when only juice consumers are considered.^17^ In Australia, 100% fruit juice consumption may increase the proportion of people meeting their daily recommended servings of fruit from 10% to 24%.^17^ It has been suggested that actively encouraging daily consumption of 100% juice in public health policies could be a practical and cost-effective way of helping populations achieve the recommended intakes for fruit and vegetables.^12^ Data from the United States suggest that consumption of 100% fruit juice is associated with improved rates of nutrient adequacy^18^ and diet quality,^19^^,^^20^ potentially through displacement of other energy-dense, nutrient-poor beverages, such as sugar-sweetened soda.^21^^,^^22^ Notably, the energy density of 100% juice is similar to low-fat milk (∼1.8 kJ/g),^23^ and in economic modeling of the Australian diet, juice had the highest nutrient density-to-cost ratio, indicating that it is a cost-effective source of nutrition.^24^

Despite these potential benefits, the place of 100% juice in dietary guidelines and models of healthy eating remains contentious, and recommendations vary by jurisdiction. The 2020–2025 Dietary Guidelines for Americans include 100% juice as part of a healthy dietary pattern and list it as a recommended primary beverage, with the recommendation that at least half of fruit intake comes from whole fruit.^25^ Other guidelines, including Australia,^22^ Spain,^26^ the Netherlands,^26^ and the United Kingdom,^27^ include fruit juice as a core food but recommend limiting servings (eg, 1 serving of 125–150 mL per day). Others, including those in Canada^28^ and France,^29^ have excluded juice from healthy eating guides based on the perceived risks of free sugars, acid, and energy levels for weight and oral health, and unfavorable direct comparisons to whole fruits. The New Zealand Ministry of Health eating and activity guidelines include fruit juices as sugary drinks and recommend limiting intake (eg, once a week),^30^ while the Brazilian guidelines recommend “unsweetened, minimally processed juices” and suggest limiting consumption of sweetened and ultra-processed juices.^31^ Recommendations, regardless of direction, center on fruit juices, with vegetable juices rarely mentioned.^22^^,^^25^^,^^26^^,^^28^^,^^29^^,^^31^ The WHO targets aim to reduce intakes of free sugars regardless of source, and therefore 100% juices are included in these limits.^32^ As such, it remains unclear how 100% fruit and vegetable juices should be treated in dietary recommendations.

The rationale surrounding the inclusion of 100% juices in dietary guidelines and models of healthy eating appears to be based on nutrient and bioactive content, which may reduce the risk of some noncommunicable diseases.^12^^,^^22^^,^^25^^,^^26^ The rationale for the exclusion of juices appears to be based on free sugars and energy content increasing the risks of other noncommunicable diseases, as well as the loss of dietary fiber relative to the whole-fruit form.^12^^,^^28^^,^^29^^,^^31^^,^^32^ However, both approaches represent a reductionist approach to nutrition, as foods and beverages are complex and cannot be reduced to single features (ie, the nutritional composition of 100% juice is more than just free sugars or low in fiber). Similarly, human health outcomes are varied and complex, and considering relationships with individual diseases in isolation may result in conflicting recommendations, despite common health conditions often occurring comorbidly. It is therefore necessary to consider the balance of evidence regarding various 100% juices to properly inform decision making with regard to policy and public health recommendations.

A previous umbrella review of the relationship between juice and health focused only on fruit juice, without consideration of vegetable juices or restriction to 100% juice.^33^ In addition, juice is often studied without the separation of sweetened juice (which contains added sugars) and fruit drinks (which only contain a small percentage of fruit juice) from 100% juice, which is undiluted and without added sugars. Therefore, we conducted an umbrella review of systematic literature reviews (SLRs) with meta-analyses (MAs) assessing the relationship between 100% fruit and/or vegetable juice consumption and human health outcomes to collate the highest-level evidence on the health effects of 100% fruit and vegetable juice.

## METHODS

This umbrella review (also known as an overview review, meta-review, or review of reviews) summarized SLRs with MAs to enable quantitative conclusions regarding the strength of evidence and dosage effect to be made, and ensured that only health outcomes with a sufficient body of evidence were considered. Systematic literature reviews with MAs were defined as follows: (1) full-text reports that labeled themselves as an SLR or MA anywhere in the text or (2) full-text reports that met the definition of an SLR with MA where it adhered to a strict scientific design based on explicit, prespecified, and reproducible methods and had a specific statistical strategy for assembling the results of multiple studies into a single quantitative estimate.^34^

This review was prospectively registered with the International Prospective Register of Systematic Reviews (PROSPERO, https://www.crd.york.ac.uk/prospero/; accessed December 1, 2022; registration no. CRD42022380588) and has been reported according to the Preferred Reporting Items for Systematic Reviews and Meta-Analyses (PRISMA) 2020 checklist (see Table S1 in the Supporting Information online)^35^ and the Preferred Reporting Items for Overviews of Reviews (PRIOR) checklist (see Table S2 in the Supporting Information online).^36^

### Eligibility criteria

The eligibility criteria according to the PICOS (Participant, Intervention/Exposure, Comparator, Outcome, Study design)^37^ format are described in Table 1. Systematic literature reviews with MAs of intervention, prospective cohort, and case-control studies on the association of 100% fruit and/or vegetable juice with health outcomes in humans of any age, sex, disease status, or geographic location were included. The definitions of 100% fruit and/or vegetable juice as (1) juice expressed directly from fruits and/or vegetables or (2), if reconstituted from concentrate, those having Brix concentrations representative to those expressed directly from the fruit or vegetable, were eligible for inclusion.^33^ Juices with any added sugars, sweeteners, herbs, spices, fortificants, or other added ingredients were excluded.

**Table 1:** PICOS criteria for inclusion of studies

Parameter	Inclusion criteria	Exclusion criteria
Participant/Population	Humans (adults and children)	Animal or in vitro
Intervention/Exposure	100% juice (fruit and/or vegetable)—including from fresh or frozen fruit/vegetables; reconstituted only if reconstituted to concentration representative of those expressed from the fruit/vegetables	Any added, sugars or other sweeteners, fortification, herbs or spices
Comparator	Control (placebo or other no juice control) or varying levels of consumption (eg, high vs low/no)	No control or comparator group; alternative intervention
Outcome	Health-related outcomes (relevant to population health) including infectious disease, noncommunicable diseases, disease risk factors or markers, immune function, cognitive function, exercise performance or recovery, and growth and development in children and adolescents	Biomarkers of juice intake, disease treatment, biomarkers not related to disease prevention.
Study design/Source	SLRs with MA	SLRs with no MA, cross-sectional studies, single-arm interventions, narrative reviews, expert opinion articles, or consensus guidelines

*Abbreviations:* MA, meta-analysis; SLR, systematic literature review.

### Search strategy

Four electronic databases were searched from inception to December 20, 2022: Medline (via PubMed), The Cochrane Library (Reviews and CENTRAL), EMBASE, and CINAHL (via EBSCO). The systematic search strategy was designed to include a combination of both controlled vocabulary (eg, MeSH [Medical Subject Headings] terms, publication type) and key words by title and abstract. The key words repeated the controlled vocabulary terms plus key words specific to the topic. The search strategy was designed in PubMed and then translated to the other databases (see Table S3 in the Supporting Information online).

### Selection process

Identified records were imported into Endnote reference management software (version 20; Clarivate Analytics, Philadelphia, PA, USA), and deduplication was performed using the automatic deduplication feature followed by a manual check to identify any remaining duplicates. The titles of the remaining references were then text-mined to identify and exclude clearly ineligible records (eg, studies conducted in mice, in vitro, or cross-sectional study designs). Excluded records identified by title text mining were checked for accuracy by a second reviewer. The remaining study records were uploaded to Covidence, a web-based systematic review software for screening (Veritas Health Innovation, Melbourne, Australia). Two reviewers (E.L.B. and M.B.) independently screened the titles and abstracts in duplicate to identify studies that potentially met the eligibility criteria. Full texts of studies were then retrieved and independently reviewed against the eligibility criteria by 2 authors (E.L.B. and M.B.). Any discrepancies were resolved by consensus or a third reviewer (C.S.). The interrater reliability between reviewers at full-text review is summarized in Table S4 (see the Supporting Information online).

Systematic literature reviews with MAs were excluded if they were unable to be translated into English via Google Translate (Google, California, USA) or manual translation by multilingual colleagues. If multiple SLRs with MAs existed on the same topic and included the same primary studies and outcomes, the degree of overlap was assessed by calculation of the corrected covered area for each type of intervention.^38^ Where high (11%–15%) or very high (>15%) overlap occurred, the SLR with MA that (1) provided the most complete description, (2) was most recent, (3) contained the most evidence, and (4) was methodologically most rigorous was included.^39^

### Data extraction

Data were extracted into summary tables by 2 independent reviewers (E.L.B. and M.B.) and cross-checked. Any discrepancies were resolved by consensus or a third reviewer (C.S.). Data extracted were as follows: study and participant characteristics, intervention/exposure (juice fruit or vegetable source, duration, and dose), comparator (type including any details of processing, duration, and dose), number of meta-analyzed studies/intervention groups, model, meta-analyzed outcome, original research study design, risk of bias as reported, sample size (intervention/case, comparator/control, and total), effect size, confidence interval, *P* value, heterogeneity, publication bias, and the Grading of Recommendations Assessment, Development and Evaluation (GRADE) quality rating (if reported). Where both fixed- and random-effects models were reported for the same data, only results from random-effects models were extracted. As few authors usually apply GRADE, the current investigators (E.L.B. and M.B.) conducted GRADE assessments for each extracted meta-analysis using the information provided in the relevant SLRs.^40^ Data were extracted into a Microsoft Excel (Microsoft Corporation, Washington, USA) spreadsheet (Microsoft Corporation, Redmond, WA, USA) by 1 researcher (E.L.B. or M.B.) and checked for accuracy by another researcher (E.L.B. or M.B.).

### Quality assessment

Each included SLR was independently assessed by 2 researchers (E.L.B. and M.B.) using the Risk of Bias in Systematic Reviews (ROBIS) tool.^41^ ROBIS has been specifically designed to assess the risk of bias in SLRs via the completion of 3 phases: (1) assess the relevance, (2) identify concerns with the review process across 4 domains (eligibility criteria, study selection, data collection/study appraisal, and synthesis/findings), and (3) judge the risk of bias. Any discrepancies were resolved by consensus or a third reviewer (C.S.).

## RESULTS

**Figure 1:**
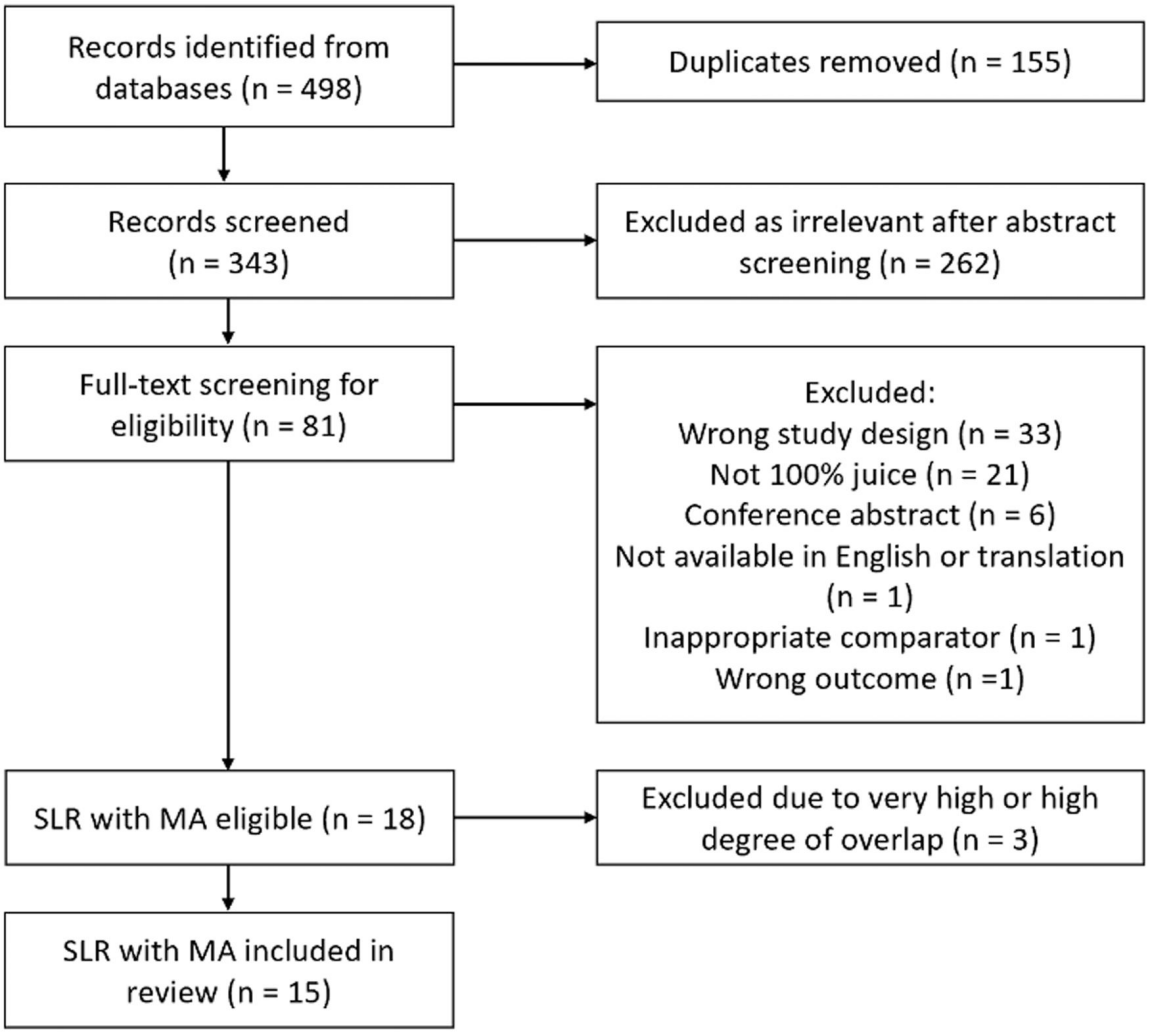
PRISMA (Preferred Reporting Items for Systematic Reviews and Meta-Analyses) flow diagram of the literature search process.

The systematic search strategy identified a total of 498 records, of which 18 SLRs with MAs were eligible for inclusion (Fig. 1). Records excluded after full-text review are listed with reasons in Table S5 (see the Supporting Information online). Three were excluded due to a high or very high degree of overlap of studies included in MAs (see Table S6 in the Supporting Information online), resulting in 15 SLRs with a total of 144 MAs included in this umbrella review. Of the extracted MAs, 51 were primary analyses and 93 were secondary subanalyses by juice type, dose, or duration of intervention.

### Characteristics of included studies

Characteristics of included studies, including ROBIS and GRADE ratings, are summarized in Tables S7 and S8, (see the Supporting Information online) and presented in more detail in the data-extraction spreadsheet published elsewhere (doi: 10.17632/6t3yx49wbs.1).^42^ The included MAs were from SLRs published between 2016 and 2022. The number of primary cohorts or interventions included in each MA ranged from 2 to 29. Of the 15 included SLRs, 5 included only prospective cohort studies, 1 included prospective cohort and case-control studies, and 9 included only intervention studies. The included MAs represented a range of doses (50–1200 mL/day) and time frames (hours to years) of juice intake (exposure). Cohort studies ranged between 4 and 20 years of follow-up, and intervention studies ranged from 2.5 hours to 18 months.

Primary MAs were conducted on 100% various fruit juices (various types combined or unspecified; n = 39), 100% pomegranate juice (n = 7), and 100% orange juice (n = 5). Secondary MAs included 87 subanalyses by dose, duration, age, and juice type (100% juices included pomegranate, orange, citrus, cranberry, grape, cherry, blackcurrant, and berry), and 6 were dose–response analyses. No studies on 100% vegetable juice met the inclusion criteria (see Table S5 in the Supporting Information online).

Participants represented the 2 binary sexes, except for the outcome of prostate cancer (males only). Systematic literature reviews contained studies mainly from Europe, Asia, and North and South America. The majority of SLRs included adults (46 primary MAs, 85 secondary analyses), with 1 SLR on adolescents (4 primary MAs) and 1 on children (1 primary MA, 2 secondary MAs). Populations included healthy participants and participants with risk factors for cardiometabolic disease and some common conditions such as type 2 diabetes.

The ROBIS quality assessment found 9 SLRs rated as having a low risk of bias, 3 as unclear, and 3 as having a high risk of bias. Using GRADE, confidence in the body of evidence ranged from very low (27 primary MAs, 79 secondary MAs), low (19 primary MAs, 13 secondary MAs), and medium (4 primary MAs, 1 secondary MA) (see Table S8 in the Supporting Information online).

Health outcomes assessed are summarized in Table 2. Health outcomes analyzed in the MAs of cohort and case-control studies were mortality (all-cause, coronary heart disease, cardiovascular disease [CVD], and stroke) and diagnosis (CVD, coronary heart disease, stroke, breast cancer, prostate cancer, type 2 diabetes, and hypertension). Health outcomes analyzed in MAs of intervention studies included liver function (aspartate aminotransferase [AST] and alanine transaminase [ALT]), body composition (body mass index [BMI], BMI *z* score, body weight, waist circumference), cardiovascular health/function (diastolic blood pressure [DBP], systolic blood pressure [SBP], total cholesterol, high-density-lipoprotein [HDL] cholesterol, low-density-lipoprotein [LDL] cholesterol, triglycerides, uric acid, intercellular adhesion molecule 1 [ICAM-1], vascular cell adhesion molecule 1 [VCAM-1], endothelial selectin [E-selectin]), metabolic health/function (blood glucose, insulin, homeostatic model assessment for insulin resistance [HOMA-IR], glycated hemoglobin [HbA1c]), and inflammation (C-reactive protein [CRP], interleukin-6 [IL-6], tumor necrosis factor alpha [TNF-α], malondialdehyde [MDA]).

**Table 2:** Summary of health outcomes assessed

Outcome category	SLRs (n)	MAs (n)			100% Juices	ROBIS	GRADE
		Primary	Secondary	Dose–response			
Mortality	5	8	—	6	Fruit (various)	Low	Very low
Body composition	2	4	14	—	Primary: fruit (various) Secondary: blackcurrant, pomegranate, mixed, grape, orange	Low/unclear	Very low
Cancer	1	3	—	—	Fruit (various)	Unclear	Very low
CVD	2	4	—	3	Fruit (various)	Low	Very low–low
CVD risk factors	5	14	37	—	Primary: fruit (various), pomegranate Secondary: pomegranate, grape, citrus, cranberry, berry, orange	Low-high	Very low–medium
Metabolic health	3	8	33	—	Primary: fruit (various) Secondary: fruit (various), pomegranate, grape, cherry, orange, cranberry, blackcurrant	Low/unclear	Very low–low
Inflammation	4	9	5	—	Primary: fruit (various), pomegranate, orange Secondary: orange	Low-high/unclear	Very low–low
Liver function	1	4	—	—	Fruit (various), orange	Low	Low–medium

“Various” refers to combined juice types, nonspecified, or juice generally.

*Abbreviations:* CVD, cardiovascular disease; GRADE, Grading of Recommendations Assessment, Development and Evaluation; MA, meta-analysis; ROBIS, Risk of Bias in Systematic Reviews; SLR, systematic literature review.

Of the 51 primary MAs, 10 (19.6%; 4 for blood pressure, 2 for vascular function, 3 for inflammation, 1 for stroke mortality) found that 100% fruit juice consumption significantly improved health outcomes, 3 (5.9%) reported an adverse relationship (1 each for CVD mortality, prostate cancer, type 2 diabetes risk), and the remaining 38 MAs (74.5%) reported no effect on health outcomes. Details of results by health outcome are provided in the following sections.

### Findings of meta-analyses of prospective cohort and case-control studies

#### Mortality

No associations were found between the highest and lowest 100% juice intakes or dose–response relationships for all-cause or coronary heart disease mortality (2–4 cohorts included in each MA),^43^^,^^44^ and no dose–response association was found for 100% juice consumption and stroke mortality^43^ (Table 3).^43–47^ However, increased risks for CVD mortality (hazard ratio [HR] = 1.2; 95% confidence interval [CI]: 1.01–1.42; MA of 2 cohorts^44^) were reported in the highest consumers (means: 237–252.75 mL/day) of 100% juice compared with the lowest consumers (Table 3). A reduced risk for stroke mortality (relative risk [RR] = 0.67; 95% CI: 0.60–0.76; MA of 3 cohorts^43^) was found among the highest 100% juice consumers. GRADE ratings were very low to low and ROBIS ratings were low (see Tables S7 and S8 in the Supporting Information online).

**Table 3:** Associations between 100% fruit juice (various) intake mortality and diagnosis in adults

Outcome	Mean follow-up (years)	No. (cohorts: participants)	Statistic^a^	CI (lower; upper)	*I^2^*	Reference
Highest vs lowest consumption						
Mortality						
All-cause	11	3: 291 725	1.00^a^	0.78; 1.29	83	Pan et al (2022)^44^
CHD	6–17	4: 141 710	0.87	0.68; 1.01	71	Zurbau et al (2020)^43^
CVD	6–16.3	2: 93 440	1.20^a^	1.01; 1.42	0	Pan et al (2022)^44^
Stroke	16	3: 128 270	0.67	0.60; 0.76	0	Zurbau et al (2020)^43^
Diagnosis						
CHD	7–19	4: 109 898	0.99	0.92; 1.07	0	Zurbau et al (2020)^43^
CVD	8–16	5: 167 879	1.00	0.93; 1.07	0	Zurbau et al (2020)^43^
Hypertension	7–20	2: 83 178	0.95	0.85; 1.07	85	Liu et al (2019)^46^
Stroke	8–16	3: 148 839	0.80	0.55; 1.15	73	Zurbau et al (2020)^43^
Prostate cancer	4–15	4: 135 359	1.03	1.01; 1.05	0	Llaha et al (2021)^45^
Colorectal cancer	4–7	3: 109 279	1.29	0.78; 2.12	0	Llaha et al (2021)^45^
Breast cancer	4–6	3: 108 837	1.13	0.93; 1.38	0	Llaha et al (2021)^45^
Type 2 diabetes^b^	5–21	13: 440 937	1.07	1.01; 1.14	50.8	Imamura et al (2015)^47^
Type 2 diabetes	5–21	13: 440 937	1.05	0.99; 1.11	57.9	Imamura et al (2015)^47^
Dose–response						
Mortality						
All-cause	11	2: 261 542	0.88^a^	0.77; 1.01	27	Pan et al (2022)^44^
CHD	6–17	4: 141 710	0.58	0.33; 1.01	71	Zurbau et al (2020)^43^
Stroke	16	3: 128 270	0.60	0.36; 1.01	0	Zurbau et al (2020)^43^
Diagnosis						
CHD	7–19	4: 109 898	0.95	0.87; 1.03	0	Zurbau et al (2020)^43^
CVD	7–19	5: 167 879	0.98	0.95; 1.02	0	Zurbau et al (2020)^43^
Stroke	8–16	3: 148 839	0.94	0.82; 1.09	73	Zurbau et al (2020)^43^

a Relative risk unless marked, those marked with “a” are hazard ratio (HR).

b Adjusted for adiposity.

*Abbreviations:* CHD, coronary heart disease; CI, confidence interval; CVD, cardiovascular disease.

#### Cancer

No associations were found between the highest and lowest 100% juice intakes and diagnosis of breast cancer or colorectal cancer (MAs of 3 cohorts each).^45^ However, increased risks for prostate cancer diagnosis (RR = 1.03; 95% CI: 1.01–1.05; MA of 4 cohorts^45^) were found in the highest consumers of 100% juice compared with the lowest consumers (Table 3). GRADE ratings were very low to low and ROBIS ratings were unclear (see Tables S7 and S8 in the Supporting Information online).

#### Cardiovascular disease

No associations were found between the highest and lowest 100% juice intakes or dose–response relationships for diagnosis of CVD (MA of 5 cohorts) or stroke incidence (MA of 3 cohorts) (Table 3).^43^^,^^44^ No associations were found between the highest and lowest 100% juice intakes and diagnosis of coronary heart disease (MA of 5 cohorts) or hypertension (MA of 2 cohorts) (Table 3).^45^^,^^46^ GRADE ratings were very low to low and ROBIS ratings were low (see Tables S7 and S8 in the Supporting Information online).

#### Metabolic health and body composition

The risk for type 2 diabetes diagnosis (RR = 1.07; 95% CI: 1.01–1.14; MA of 13 cohorts^47^) was increased in the highest 100% juice consumers compared with the lowest when models were adjusted for adiposity; no effects were reported without this adjustment. In children, there were no associations found between BMI *z* score and 100% juice consumption in both primary analyses (MA of 8 comparisons, representing 28 037 participants, including children aged 1–18 y), in energy-adjusted and nonadjusted models.^48^ In age-stratified secondary subanalyses, 100% juice consumption was associated with a small amount of non–clinically significant weight gain in the models not adjusted for energy consumption for ages 1–6 years only.^48^ GRADE ratings were very low to low and ROBIS ratings were low/unclear (see Tables S7 and S8 in the Supporting Information online).

### Findings of meta-analyses of intervention studies

#### Cardiovascular disease risk factors

Three SLRs with 11 primary MAs and 37 secondary MAs (juice type, dose, and duration) investigated CVD-related parameters, including DBP, SBP, blood uric acid levels, flow-mediated dilation, pulse-wave velocity, total cholesterol, triglycerides, HDL cholesterol, and LDL cholesterol (Table 4).^49–52^

**Table 4:** Results from primary MAs of 100% fruit juice interventions for CVD-related outcomes

Outcome	Duration	Dose (mL/day)	No. of interventions: participants	Statistic^a^	CI (lower; upper)	*I^2^*	Reference
DBP (mmHg)^b^	1–16 wk	100–1000	26: 1225	–1.68	–2.94; –0.43	31.4	D’Elia et al (2021)^49^
DBP (mmHg)^c^	2 wk–18 mo	50–500	8: 574	–2.01	–3.71; –0.31	NR	Sahebkar et al (2017)^50^
SBP (mmHg)^b^	1–16 wk	100–1000	26: 1225	–3.14	–4.43; -1.85	0	D’Elia et al (2021)^49^
SBP (mmHg)^c^	2 wk–18 mo	50–500	8: 574	–4.96	–7.67; -2.25	NR	Sahebkar et al (2017)^50^
Uric acid (mg/dL)^b^	1–12 wk	120–1200	8: 390	–0.28	–0.43; –0.13	11.1	Ayoub-Charette et al (2021)^51^
FMD (%)^b^	1–6 wk	480–1000^d^	5: 174	2.10	1.14; 3.06	0	D’Elia et al (2021)^49^
PWV (m/s)^b^	2–8 wk	250–500^d^	6: 256	–0.03	–0.41; 0.35	55.7	D’Elia et al (2021)^49^
Total cholesterol (mg/dL)^b^	1–16 wk	120–1000	29: 1180	–3.15	–6.43; 0.13	0	D’Elia et al (2021)^49^
Triglycerides (mg/dL)^b^	1–16 wk	120–750	26: 1049	–0.65	–5.83; 4.52	0	D’Elia et al (2021)^49^
HDL cholesterol (mg/dL)^b^	1–16 wk	120–750	25: 1007	0.43	–0.72; 1.59	9	D’Elia et al (2021)^49^
LDL cholesterol (mg/dL)^b^	1–13 wk	120–750	23: 880	0.29	–2.62; 3.2	0	D’Elia et al (2021)^49^
ICAM-1^c^	2–12 wk	150–250	4: 125	–0.42	–1.01; 0.17	0	Asgary et al (2021)^52^
E-selectin^c^	2–12 wk	150–250	4: 125	–0.21	–1.62; 1.21	86	Asgary et al (2021)^52^
VCAM-1^c^	2–12 wk	150–250	4: 125	–0.2	–1.95; 1.95	86.8	Asgary et al (2021)^52^

a Mean difference (intervention vs control).

b 100% juice (various types).

c 100% pomegranate juice.

d Dose calculated using 7 mL/kg/d.

*Abbreviations:* CI, confidence interval; CVD, cardiovascular disease; DBP, diastolic blood pressure; E-selectin, endothelial selectin; FMD, flow-mediated dilation; HDL, high-density lipoprotein; ICAM, intercellular adhesion molecule; LDL, low-density lipoprotein; MA, meta-analysis; NR, not reported; PWV, pulse-wave velocity; SBP, systolic blood pressure; VCAM, vascular cell adhesion molecule.

In a large MA (n = 1225 from 26 intervention trials) of 100% fruit juice, clinically and statistically significant reductions in DBP (–1.68 mmHg; 95% CI: –2.94, –0.43) and SBP (–3.14 mmHg; 95% CI: –4.43, –1.85) were found in doses of 100–1000 mL/day for 1–16 weeks.^49^ In secondary MAs by juice type, only 100% pomegranate juice (1–13 wk; 240–500 mL) had an impact on DBP and SBP.^49^ Findings were supported by an earlier primary MA of 8 intervention trials of 100% pomegranate juice that found reductions in DBP (–2.01 mmHg; 95% CI: –3.71, –0.31) and SBP (–4.96 mmHg; 95% CI: –7.67, –2.25) with 50–500 mL per day over 2 weeks to 18 months.^50^ Secondary MAs showed improvements in blood pressure regardless of dose (both above and below 240 mL/day) and only in studies longer than 12 weeks.^50^ GRADE ratings were very low to low and ROBIS ratings were high/unclear (see Tables S7 and S8 in the Supporting Information online).

Primary MAs on 100% fruit juice also found improvements in uric acid levels^51^ and clinically relevant improvements in flow-mediated dilation^49^ (Table 4). The 100% juice interventions did not influence blood lipid measures (total cholesterol, triglycerides, HDL cholesterol, and LDL cholesterol) in primary (1 primary MA per outcome) (Table 4) or secondary (5 secondary MAs per outcome, with the exception of cholesterol with 7 secondary MAs) MAs. In adolescents, no associations were found between 100% juice consumption and markers of vascular function (ICAM-1, VCAM-1, and E-selectin) in 3 primary MAs (150–250 mL/day for 2–12 wks; MA of 4 intervention trials) (Table 4). GRADE ratings were low and ROBIS ratings were unclear (see Tables S7 and S8 in the Supporting Information online).

#### Inflammation

Conflicting results were found within 9 primary MAs on inflammatory measures (IL-6, CRP, MDA, TNF-α) (Table 5).^52–55^ Two primary MAs reported that 100% pomegranate juice in adolescents (50–240 mL/day for 2 wk to 18 mo; MA of 5 intervention trials)^52^ and 100% orange juice in adults (240–1000 mL/day for 5 h to 4 wk; MA of 4 intervention trials)^53^ reduced IL-6 levels (–1.07 pg/mL; 95% CI: –1.9, –0.19; and –1.51 pg/mL; 95% CI: –2.31, –0.70, respectively). In secondary MAs of IL-6, 100% orange juice in adults by both acute (hours) and chronic (weeks) intervention maintained significant differences.^53^ However, another primary MA of 100% fruit juice (various types) in adults found no association with IL-6 levels.^54^ CRP levels were significantly reduced in 1 primary MA (of 2 intervention trials) of substitution trials (those where the juice intervention was energy matched to the controls) of 100% juice in adults (various types; interventions 500 mL/day for 2 wk),^54^ but not in the MA of 12 addition trials (those where the juice intervention provided additional energy compared with the controls; 100–700 mL/day for 2–13 wk^54^) (Table 5). In a secondary MA, CRP levels were reduced in chronic (weeks) interventions with 100% orange juice, but no changes were reported in the primary MA of 100% orange juice^53^ or 100% pomegranate juice.^55^ In primary MAs, 100% fruit juice (various) interventions did not impact TNF-α levels (4–9 wk of intervention at 200–700 mL; MA of 3 intervention trials)^54^ and 100% orange juice interventions did not impact MDA levels (2.5 h–12 wk of intervention at 500 mL; MA of 3 intervention trials), with results remaining unchanged in a subanalysis excluding the trials of less than 4 weeks.^53^ GRADE ratings were very low to medium and ROBIS ratings were low–high/unclear (see Tables S7 and S8 in the Supporting Information online).

**Table 5:** Results from primary MAs of 100% fruit juice interventions for inflammation-related outcomes

Outcome	Duration	Dose (mL/day)	No. of interventions: participants	Statistic^a^	CI (lower; upper)	*I^2^*	Reference
IL-6 (pg/mL)^b^	5 h–4 wk	240–1000	4: 176	–1.51	–2.31; –0.70	23.0	Cara et al. (2022)^55^
IL-6 (pg/mL)^c^	2–13 wk	150–700	4: 180	–3.01	–6.91; 0.88	74.1	Qi et al (2022)^54^
IL-6 (pg/mL)^d^	2–48 wk	50–240	7: 301	–1.07^g^	–1.9; –0.19	87.6	Asgary et al (2021)^52^
CRP (mg/L)^e^	2 wk	500	2: 150	–1.09	–0.17; -2.01	0.0	Qi et al (2022)^54^
CRP (mg/L)^f^	2–13 wk	100–700	12: 506	–0.12	–0.53; 0.30	49.3	Qi et al (2022)^54^
CRP (mg/L)^d^	2 wk–18 mo	150–250^e^	5: 432	–0.22	–0.45; 0.01	NR	Sahebkar et al (2016)^55^
CRP (mg/L)^b^	1–12 wk	500–1000^e^	4: 138	–0.58	–1.22; 0.05	78.8	Cara et al (2022)^53^
TNF-α (pg/L)^f^	4–9 wk	200–700	3: 176	–0.66	–2.66; 1.35	95.6	Qi et al (2022)^54^
MDA (µmol/L)^b^	2.5 h–12 wk	500	3: 127	–0.06	–0.19; 0.08	45.3	Cara et al (2022)^53^

a Mean difference unless marked.

b 100% orange juice.

c 100% juice (various types).

d 100% pomegranate juice.

e 100% juice (various types) substitution trials.

f 100% juice (various types) addition trials.

g Standardized mean difference.

*Abbreviations:* CI, confidence interval; CRP, C-reactive protein; IL, interleukin; MA, meta-analysis; MDA, malondialdehyde; NR, not reported; TNF-α, tumor necrosis factor alpha.

#### Metabolic health

Two SLRs included 8 primary MAs on metabolic health–related measures for 100% fruit juice (various types, 120–595 mL/day) interventions (2 MAs each for insulin with 11 intervention trials each, blood glucose with 16–23 intervention trials each, HbA1c with 3 intervention trials each, HOMA-IR with 7–11 intervention trials each), with 32 secondary MAs by juice type, dose, and duration of intervention (see Table S9 in the Supporting Information online). No relationships were found between metabolic health parameters and 100% juice consumption in primary MAs^49^^,^^56^ (see Table S9 in the Supporting Information online). However, fasting blood glucose and HOMA-IR levels were reduced in secondary MAs when the shortest interventions were included (<7 wk),^56^ while no differences were found in longer interventions. HOMA-IR was improved in a secondary MA of 100% pomegranate juice only, with no effects reported in other juice types.^56^ GRADE ratings were very low to low and ROBIS ratings were low/unclear (see Tables S7 and S8 in the Supporting Information online).

#### Body composition

One SLR with 3 primary MAs^49^ assessed the impact of 100% fruit juice interventions on body-composition measures, with no associations found in either primary MAs (120–1000 mL/day for 1–16 wk) or secondary MAs by juice type for BMI (MA of 13 intervention trials), waist circumference (MA of 10 intervention trials), and body-weight measures (MA of 20 intervention trials) in adults (see Table S10 in the Supporting Information online). GRADE ratings were very low and ROBIS ratings were unclear (see Tables S7 and S8 in the Supporting Information online).

#### Liver function

No associations were found between 100% fruit juice (various types, 120–500 mL/day) and liver function markers (ALT and AST, 1 MA each for addition and 1 MA each for substitution with 2–7 intervention trials in each MA; 120–500 mL/day for 28 days to 12 wk) (see Table S11 in the Supporting Information online),^57^ regardless of study design (addition or substitution trials). GRADE ratings were low to medium and ROBIS ratings were low (see Tables S7 and S8 in the Supporting Information online).

## DISCUSSION

This umbrella review has synthesized the diverse body of quantitative evidence on the health effects of 100% juice. Results from MAs were available for a broad range of doses (50–1200 mL/day, including several interventions using relatively high doses of >500 mL/day) and durations (hours to multiple years). Results from meta-analyses were available for several health outcomes of key relevance to population health, including mortality, CVD, metabolic health, body composition, cancer, and inflammation.

On balance, when multiple juice types, doses, durations of exposure, and health outcomes were considered, limited potential harms associated with 100% juice consumption were identified, with the majority of the available evidence (including MAs from both intervention and prospective cohort studies) being neutral (neither positive or negative). Improved cardiovascular and inflammatory health measures were seen in MAs of intervention studies and, for cardiovascular outcomes, effect sizes were of clinical relevance, while this is more difficult to assess for inflammatory markers. Unfavorable associations were found between 100% juice consumption and type 2 diabetes risk (RR = 1.07), CVD mortality (HR = 1.20), and prostate cancer risk (RR = 1.03), with the totality of this evidence from MAs of cohort studies. The mismatch between intervention and observational studies for CVD-related findings may reflect differences in time frames or confounding in observational trials. This may mean that the true effect size is larger or smaller, with the confidence level being low due to the small number of cohorts included in the meta-analyses. However, these were notably very small effect sizes, even for nutritional epidemiology where smaller RRs are more common than in other epidemiological fields.^58^

Weak associations from cohort studies may be meaningful if supported by randomized controlled trials or laboratory studies.^58^ Interestingly, higher 100% juice consumption was associated with an increased risk for diagnosis of type 2 diabetes and CVD in prospective cohort studies but was not linked to risk factors for these conditions in the meta-analyses of intervention studies. No associations were found for type 2 diabetes risk factors (eg, blood glucose levels and insulin resistance), with some protective effects found for CVD risk factors (eg, blood pressure levels). The polarity and diversity of findings regarding the relationship between 100% juice and health may be explained by differences in primary study methods, with the majority of favorable findings being reported in intervention studies and unfavorable findings being only from prospective cohort studies. Of the 12 included primary MAs of prospective cohort studies, 3 MAs (25%) showed risk and 1 MA (8%) showed benefit, whereas 8 MAs (66%) showed null effects. The single MA that combined cohort and case-control studies showed no associations. Of the 38 primary MAs that included intervention studies, 9 (23%) showed benefit, none showed risks, and the remainder (77%) showed neutral effects, including studies of relatively high daily intakes of more than 500 mL per day.

While prospective cohort studies are the highest level of primary evidence for disease etiology,^59^ data-collection methods, such as food-frequency questionnaires and food recalls, may not adequately measure intake of 100% juices, as juice is a beverage product with significant diversity, and these methods, not designed to capture intakes of specific foods or beverages, are more likely to be used in cohort studies. It is possible that the “juices” captured by these dietary data-collection methods are not solely 100% juice, including fruit drinks or sweetened juices, despite being reported as 100% juice.^60^^,^^61^ Intervention studies, while only able to consider relatively short-term health outcomes, have the benefit of 100% juice intake being tightly controlled and thus more reliable. It is also possible that the favorable results seen in intervention trials are more pronounced due to the inclusion of participants with a background of low fruit and/or vegetable consumption. The complex and multifactorial nature of chronic disease risk (eg, genetics and additional lifestyle risk factors) may additionally help explain the differences in findings between complex outcomes (as measured in prospective cohort studies) and simple outcomes (as measured in intervention trials). However, it is important to note that, while intervention studies are able to be randomized, they are restricted to short-term outcomes, and may not adequately capture changes or impacts of modulating factors seen over the longer term. Conversely, prospective cohort studies are able to represent long-term outcomes in large cohorts but do not readily allow for precision analyses regarding individual foods or beverages consumed, subpopulations based on genetics, or other potentially modifying factors.

The positive impact of 100% juice consumption on specific health outcomes relevant to population health may be explained by multiple potential mechanisms, including the vitamin, mineral, and bioactive compound content of 100% juice. Data from epidemiological and animal studies show that high potassium intake can reduce blood pressure, arterial stiffness, and associated vascular and organ damage,^49^^,^^62–64^ with 100% fruit and/or vegetable juice being an affordable, accessible, and palatable source of potassium.^13^^,^^65^ Furthermore, there is significant evidence linking color-associated compounds to health benefits, and juices are readily available in a variety of colors with bioactive pigments maintained.^7^ Many bioactive compounds, including polyphenols such as hesperidin and naringenin, are highly bioavailable in some juices and may contribute to beneficial cardiovascular and inflammatory effects via modulation of nitric oxide bioavailability and related pathways,^7^^,^^49^^,^^66^^,^^67^ with polyphenol intake linked to improved blood pressure outcomes and higher plasma nitric oxide levels.^65^^,^^66^ The antioxidant activity of bioactive compounds (such as flavonoids, anthocyanins, and carotenoids) and vitamins (such as vitamins C and A) may also contribute to improved cardiovascular and inflammatory outcomes.^49^^,^^67^ Juices containing folate may benefit health via reduction in homocysteine levels, high levels of which can damage blood vessels.^68^ However, all of these features can vary by juice type, and differing juices included in studies may impact results.

Oral health concerns are commonly cited as reasons to recommend restricting or excluding juice intake.^22^^,^^26^^,^^28^^,^^29^^,^^31^ However, no MAs on the relationship between dental caries and other oral health outcomes were eligible for this review when the 100% juice inclusion criteria were applied. This suggests that previous reviews (eg,^21^) may have included data for sweetened juices or other non-100% juices, making the findings not specific to 100% juices. However, a recent SLR without MA on 100% fruit juice and dental health found that the existing data of 5 independent prospective cohort studies and 9 randomized controlled trials was inconclusive regarding the risks, with the randomized controlled trial data demonstrating harm using intra-oral devices lacking normal plaque or saliva action and conditions not representative of normal juice consumption.^69^ Acidic beverages are often associated with tooth erosion^70^; however, it has been argued that assigning risk to dietary factors in this context is inappropriate due to the complexity of behavioral factors involved in oral hygiene and risk for tooth erosion and caries^71^ and the modifying effects of toothpastes.^72–74^ As such, SLRs with MA investigating the relationships between oral health outcomes and 100% juice consumption are still needed.

One hundred percent juice is well known to be lower in dietary fiber relative to whole fruit,^75^ which, as an isolated comparison, suggests that 100% juice would adversely impact satiety and therefore energy intake and weight management, and this is regularly cited as a reason to recommend limiting intake.^22^^,^^26^^,^^28^^,^^29^^,^^31^ Satiety was not included in the present review, as it is not a health outcome directly; however, included intervention studies for adults on body weight, BMI, and waste circumference (with 10–20 interventions per MA) did not find any relationship between 100% juice consumption and body-composition measures, noting that doses ranged from 120 to 1000 mL/day for between 1 and 16 weeks. It is important to note that these included relatively small numbers of participants with MA pools of 411–975 participants, depending on the metric. Where subanalyses were available by juice type, this led to a separation of duration and dose as a consequence, and even the high doses and long durations (eg, 1000 mL/day over 6 wk or 480 mL/day over 12–16 wk) still had no significant associations. Studies in children were cohort studies based on BMI *z* score and were considerably larger. Importantly, consideration of fiber content alone on satiety does not consider the context of 100% juice in the daily diet, nor the contribution of other features in 100% juice. For example, an intervention study on the timing of juice consumption found weight gain when juice (20% of daily energy requirement) was consumed between meals, but weight loss when the same amount was consumed with meals.^75^ In a study of grape juice, there was no weight gain seen over 12 weeks in those consuming the juice (compared with a no-beverage control); however, there was weight gain in those consuming an energy matched, polyphenol-free beverage,^76^ suggesting that polyphenols impact satiety or energy metabolism.

While this umbrella review was conducted following best-practice protocols and provides a broad overview of quantitative analysis from existing MAs, several limitations need to be considered when interpreting the findings. The protocol was designed to include only 100% juices. However, during review and screening, it was found that many SLRs that purported to include only 100% juice, in fact included primary studies where this restriction was not applied, leading to the inclusion of fruit drinks and sweetened and/or fortified juices. While all efforts were made to ensure the inclusion of only data from studies of 100% juices, it is possible that differences in definitions, descriptions, and interpretations may impact the inclusion of studies in this synthesis. Studies did not include sufficient descriptions to separate juices with pulp from filtered juices.

The application of GRADE revealed many of the included MAs to be of very low or low-quality level. This suggests a need for studies with high-quality design and reporting to better inform decision making. However, a large body of evidence scoring low quality on GRADE is not unusual in nutrition research as the tool relies heavily on the clinical intervention paradigm.^77^ Findings may be limited by including only SLRs with MA; while this is justified based on MAs providing quantitative synthesis and demonstrating a sufficient body of evidence to allow for MA to be conducted, additional juices (such as vegetable juices) and additional health outcomes may be captured in SLRs without MA. This suggests that additional research, including more primary studies and more SLRs with MA, particularly those with dose–response analyses, are required to broaden the umbrella able to be reviewed in the future.

Importantly, this review sought to investigate both 100% fruit and vegetable juices. However, no MAs on 100% vegetable juice that met the inclusion criteria were available. While fruit juices are more commonly produced and consumed, vegetable juices also have beneficial micronutrients and bioactive compounds, and vegetable consumption is even lower than fruit consumption at a population level. As such, it is important to consider the potential for vegetable juices to be incorporated into models of healthy eating to address this gap. Future studies should investigate this possibility. The types of juices included in primary MAs and subanalyses may also need to be considered. Juices are often used in research studies as a delivery method for fruit nutrients and bioactive compounds to address research questions surrounding the fruit and its properties while extending shelf-life, enhancing palatability, and reducing variability in the controlled administration. However, this leads to 2 potential limitations: (1) an imbalance in the types of 100% fruit juices studied, with the predominant juice types (eg, pomegranate, grape, and cherry juices) not necessarily reflecting the most commonly consumed juices in the broader population, and (2) a potential merging of commercial, processed juices with juices prepared fresh or under small-batch conditions, leading to potential variance in pulp (and therefore dietary fiber) content and other nutrient contents due to heat/pressure treatment and storage differences. Meta-analyses can also combine studies of different varieties (eg, different types of oranges used to make orange juice), meaning results may be variable and not apply to all 100% juice types.

## CONCLUSION

In conclusion, the balance of evidence does not justify recommendations that exclude or limit 100% juice intake in models of healthy eating and dietary guidelines; however, caution may be warranted for specific subgroups or individuals, depending on risk factors and circumstances. Results include a broad range of doses and durations that have been studied using randomized controlled trials, including several intervention doses that are relatively high (500–1200 mL/day). As such, dietary guidelines for restriction or avoidance of 100% juice are not supported by the current available evidence and may have unintended consequences on population nutrient and bioactive intakes. However, additional well-designed studies are needed focusing on key health outcomes, commonly consumed 100% juice types, as well as 100% vegetable juices. Furthermore, future intervention studies need to be designed to consider actual dietary intakes, and the displacement effect of added juices and cohort studies need to be better designed to separate 100% juices from other juices and fruit drinks.

Importantly, the multiple potential beneficial effects detected at low to moderate levels (50–240 mL/day) of 100% juice consumption can provide exposure to beneficial nutrients and bioactive compounds, without the risks associated with excess consumption of free sugars and calories.^49^ At these low and moderate intake levels, 100% juice can be incorporated into a healthy, balanced diet. When multiple key health outcomes are considered, the balance of evidence suggests that views of 100% juice products formed only on single-nutrient features may be oversimplified and not evidence based, and the inclusion of 100% juice as a core food may be warranted when the potential benefits of the whole beverage are considered. The evidence presented here reflects potential health benefits for 100% fruit juice, regardless of its properties relative to whole fruits; however, the body of evidence is not yet conclusive.

## Supplementary Material

nuae036_Supplementary_Data

## Supporting Information

The following Supporting Information is available through the online version of this article at the publisher’s website.

*Table S1*  **PRISMA checklist**

*Table S2* **PRIOR checklist**

*Table S3* **Search strategy**

*Table S4* **Interrater reliability between reviewers at full-text review**

*Table S5* **Studies excluded after full-text review**

*Table S6* **SLRs excluded due to overlap**

*Table S7* **ROBIS summary**

*Table S8* **GRADE**

*Table S9* **Results from primary MAs of 100% fruit juice interventions for metabolic health**

*Table S10* **Results from primary MAs of 100% fruit juice interventions for body composition**

*Table S11*  **Results from primary MAs of 100% fruit juice interventions for liver function**
