# Supplementary material for: Health effects of drinking 100% juice: an umbrella review of systematic reviews with meta-analyses
Source: Nutr Rev. 2024 Apr 29;83(2):e722–35. doi: 10.1093/nutrit/nuae036 (PMC11723140; doi:10.1093/nutrit/nuae036)
Supplement: nuae036_Supplementary_Data [file nuae036_supplementary_data.docx]

Supplement Juice Manuscript

**Supplementary Table S1**. PRISMA checklist

| **Section and Topic** | **Item #** | **Checklist item** | **Location where item is reported** |
| --- | --- | --- | --- |
| **TITLE** | | |  |
| Title | 1 | Identify the report as a systematic review. | Umbrella Title and in methods |
| **ABSTRACT** | | |  |
| Abstract | 2 | See the PRISMA 2020 for Abstracts checklist. | Yes |
| **INTRODUCTION** | | |  |
| Rationale | 3 | Describe the rationale for the review in the context of existing knowledge. | Page 2 and 3 |
| Objectives | 4 | Provide an explicit statement of the objective(s) or question(s) the review addresses. | Page 3 |
| **METHODS** | | |  |
| Eligibility criteria | 5 | Specify the inclusion and exclusion criteria for the review and how studies were grouped for the syntheses. | Page 4 |
| Information sources | 6 | Specify all databases, registers, websites, organisations, reference lists and other sources searched or consulted to identify studies. Specify the date when each source was last searched or consulted. | Page 4 |
| Search strategy | 7 | Present the full search strategies for all databases, registers and websites, including any filters and limits used. | Pages 4-5 |
| Selection process | 8 | Specify the methods used to decide whether a study met the inclusion criteria of the review, including how many reviewers screened each record and each report retrieved, whether they worked independently, and if applicable, details of automation tools used in the process. | Pages 4-5 |
| Data collection process | 9 | Specify the methods used to collect data from reports, including how many reviewers collected data from each report, whether they worked independently, any processes for obtaining or confirming data from study investigators, and if applicable, details of automation tools used in the process. | Pages 4-5 |
| Data items | 10a | List and define all outcomes for which data were sought. Specify whether all results that were compatible with each outcome domain in each study were sought (e.g. for all measures, time points, analyses), and if not, the methods used to decide which results to collect. | Pages 4-6 |
|  | 10b | List and define all other variables for which data were sought (e.g. participant and intervention characteristics, funding sources). Describe any assumptions made about any missing or unclear information. | Pages 4-5 |
| Study risk of bias assessment | 11 | Specify the methods used to assess risk of bias in the included studies, including details of the tool(s) used, how many reviewers assessed each study and whether they worked independently, and if applicable, details of automation tools used in the process. | Page 6 |
| Effect measures | 12 | Specify for each outcome the effect measure(s) (e.g. risk ratio, mean difference) used in the synthesis or presentation of results. | Page 6 |
| Synthesis methods | 13a | Describe the processes used to decide which studies were eligible for each synthesis (e.g. tabulating the study intervention characteristics and comparing against the planned groups for each synthesis (item #5)). | Pages 4-6 |
|  | 13b | Describe any methods required to prepare the data for presentation or synthesis, such as handling of missing summary statistics, or data conversions. | Pages 4-6 |
|  | 13c | Describe any methods used to tabulate or visually display results of individual studies and syntheses. | Pages 4-6 |
|  | 13d | Describe any methods used to synthesize results and provide a rationale for the choice(s). If meta-analysis was performed, describe the model(s), method(s) to identify the presence and extent of statistical heterogeneity, and software package(s) used. | Pages 4-6 |
|  | 13e | Describe any methods used to explore possible causes of heterogeneity among study results (e.g. subgroup analysis, meta-regression). | Pages 4-6 |
|  | 13f | Describe any sensitivity analyses conducted to assess robustness of the synthesized results. | N/A |
| Reporting bias assessment | 14 | Describe any methods used to assess risk of bias due to missing results in a synthesis (arising from reporting biases). | Pages 4-6 |
| Certainty assessment | 15 | Describe any methods used to assess certainty (or confidence) in the body of evidence for an outcome. | Pages 4-6 |
| **RESULTS** | | |  |
| Study selection | 16a | Describe the results of the search and selection process, from the number of records identified in the search to the number of studies included in the review, ideally using a flow diagram. | Page 7 |
|  | 16b | Cite studies that might appear to meet the inclusion criteria, but which were excluded, and explain why they were excluded. | Supplement |
| Study characteristics | 17 | Cite each included study and present its characteristics. | Data repository |
| Risk of bias in studies | 18 | Present assessments of risk of bias for each included study. | Supplement |
| Results of individual studies | 19 | For all outcomes, present, for each study: (a) summary statistics for each group (where appropriate) and (b) an effect estimate and its precision (e.g. confidence/credible interval), ideally using structured tables or plots. | Pages 8-15 |
| Results of syntheses | 20a | For each synthesis, briefly summarise the characteristics and risk of bias among contributing studies. | Pages 8-15 |
|  | 20b | Present results of all statistical syntheses conducted. If meta-analysis was done, present for each the summary estimate and its precision (e.g. confidence/credible interval) and measures of statistical heterogeneity. If comparing groups, describe the direction of the effect. | Pages 8-15 |
|  | 20c | Present results of all investigations of possible causes of heterogeneity among study results. | N/A |
|  | 20d | Present results of all sensitivity analyses conducted to assess the robustness of the synthesized results. | N/A |
| Reporting biases | 21 | Present assessments of risk of bias due to missing results (arising from reporting biases) for each synthesis assessed. | Supplement |
| Certainty of evidence | 22 | Present assessments of certainty (or confidence) in the body of evidence for each outcome assessed. | Supplement |
| **DISCUSSION** | | |  |
| Discussion | 23a | Provide a general interpretation of the results in the context of other evidence. | Page 16 |
|  | 23b | Discuss any limitations of the evidence included in the review. | Page 17-18 |
|  | 23c | Discuss any limitations of the review processes used. | Page 17-18 |
|  | 23d | Discuss implications of the results for practice, policy, and future research. | Page 18 |
| **OTHER INFORMATION** | | |  |
| Registration and protocol | 24a | Provide registration information for the review, including register name and registration number, or state that the review was not registered. | Page 4 |
|  | 24b | Indicate where the review protocol can be accessed, or state that a protocol was not prepared. | Page 4 |
|  | 24c | Describe and explain any amendments to information provided at registration or in the protocol. | N/A |
| Support | 25 | Describe sources of financial or non-financial support for the review, and the role of the funders or sponsors in the review. | Coverpage |
| Competing interests | 26 | Declare any competing interests of review authors. | Coverpage |
| Availability of data, code and other materials | 27 | Report which of the following are publicly available and where they can be found: template data collection forms; data extracted from included studies; data used for all analyses; analytic code; any other materials used in the review. | Page 7 |

**Supplementary Table S2**. PRIOR checklist

| Section topic | Item No | Item | Location where item is reported |
| --- | --- | --- | --- |
| **Title** | | |  |
| Title | 1 | Identify the report as an overview of reviews. | Identified as umbrella review in title, overview of reviews listed as study type on page 4 |
| **Abstract** | | |  |
| Abstract | 2 | Provide a comprehensive and accurate summary of the purpose, methods, and results of the overview of reviews. | Page 1 |
| **Introduction** | | |  |
| Rationale | 3 | Describe the rationale for conducting the overview of reviews in the context of existing knowledge. | Page 2 & 3 |
| Objectives | 4 | Provide an explicit statement of the objective(s) or question(s) addressed by the overview of reviews. | Page 3 |
| **Methods** | | |  |
| Eligibility criteria | 5a | Specify the inclusion and exclusion criteria for the overview of reviews. If supplemental primary studies were included, this should be stated, with a rationale. | Page 4 |
|  | 5b | Specify the definition of “systematic review” as used in the inclusion criteria for the overview of reviews. | Page 4 |
| Information sources | 6 | Specify all databases, registers, websites, organisations, reference lists, and other sources searched or consulted to identify systematic reviews and supplemental primary studies (if included). Specify the date when each source was last searched or consulted. | Page 5 |
| Search strategy | 7 | Present the full search strategies for all databases, registers and websites, such that they could be reproduced. Describe any search filters and limits applied. | Page 5 and Supplementary Tables |
| Selection process | 8a | Describe the methods used to decide whether a systematic review or supplemental primary study (if included) met the inclusion criteria of the overview of reviews. | Pages 4 and 5 |
|  | 8b | Describe how overlap in the populations, interventions, comparators, and/or outcomes of systematic reviews was identified and managed during study selection. | Page 5 |
| Data collection process | 9a | Describe the methods used to collect data from reports. | Page 6 |
|  | 9b | If applicable, describe the methods used to identify and manage primary study overlap at the level of the comparison and outcome during data collection. For each outcome, specify the method used to illustrate and/or quantify the degree of primary study overlap across systematic reviews. | Page 5 |
|  | 9c | If applicable, specify the methods used to manage discrepant data across systematic reviews during data collection. | Page 6 |
| Data items | 10 | List and define all variables and outcomes for which data were sought. Describe any assumptions made and/or measures taken to identify and clarify missing or unclear information. | Page 4-6 |
| Risk of bias assessment | 11a | Describe the methods used to assess risk of bias or methodological quality of the included systematic reviews. | Page 6 |
|  | 11b | Describe the methods used to collect data on (from the systematic reviews) and/or assess the risk of bias of the primary studies included in the systematic reviews. Provide a justification for instances where flawed, incomplete, or missing assessments are identified but not reassessed. | Page 5-6 |
|  | 11c | Describe the methods used to assess the risk of bias of supplemental primary studies (if included). | Page 5-6 |
| Synthesis methods | 12a | Describe the methods used to summarise or synthesise results and provide a rationale for the choice(s). | Page 6 |
|  | 12b | Describe any methods used to explore possible causes of heterogeneity among results. | Page 6 |
|  | 12c | Describe any sensitivity analyses conducted to assess the robustness of the synthesised results. | N/A (no qualitative synthesis) |
| Reporting bias assessment | 13 | Describe the methods used to collect data on (from the systematic reviews) and/or assess the risk of bias due to missing results in a summary or synthesis (arising from reporting biases at the levels of the systematic reviews, primary studies, and supplemental primary studies, if included). | Page 5-6 |
| Certainty assessment | 14 | Describe the methods used to collect data on (from the systematic reviews) and/or assess certainty (or confidence) in the body of evidence for an outcome. | Page 5-6 |
| **Results** | | |  |
| Systematic review and supplemental primary study selection | 15a | Describe the results of the search and selection process, including the number of records screened, assessed for eligibility, and included in the overview of reviews, ideally with a flow diagram. | Page 7 |
|  | 15b | Provide a list of studies that might appear to meet the inclusion criteria, but were excluded, with the main reason for exclusion. | Supplement |
| Characteristics of systematic reviews and supplemental primary studies | 16 | Cite each included systematic review and supplemental primary study (if included) and present its characteristics. | Data repository |
| Primary study overlap | 17 | Describe the extent of primary study overlap across the included systematic reviews. | Page 7 |
| Risk of bias in systematic reviews, primary studies, and supplemental primary studies | 18a | Present assessments of risk of bias or methodological quality for each included systematic review. | Supplement |
|  | 18b | Present assessments (collected from systematic reviews or assessed anew) of the risk of bias of the primary studies included in the systematic reviews. | Supplement |
|  | 18c | Present assessments of the risk of bias of supplemental primary studies (if included). | Data repository |
| Summary or synthesis of results | 19a | For all outcomes, summarise the evidence from the systematic reviews and supplemental primary studies (if included). If meta-analyses were done, present for each the summary estimate and its precision and measures of statistical heterogeneity. If comparing groups, describe the direction of the effect. | Pages 7-16 and Supplement |
|  | 19b | If meta-analyses were done, present results of all investigations of possible causes of heterogeneity. | N/A |
|  | 19c | If meta-analyses were done, present results of all sensitivity analyses conducted to assess the robustness of synthesised results. | N/A |
| Reporting biases | 20 | Present assessments (collected from systematic reviews and/or assessed anew) of the risk of bias due to missing primary studies, analyses, or results in a summary or synthesis (arising from reporting biases at the levels of the systematic reviews, primary studies, and supplemental primary studies, if included) for each summary or synthesis assessed. | Supplement and data repository |
| Certainty of evidence | 21 | Present assessments (collected or assessed anew) of certainty (or confidence) in the body of evidence for each outcome. | Supplement and data repository |
| **Discussion** | | |  |
| Discussion | 22a | Summarise the main findings, including any discrepancies in findings across the included systematic reviews and supplemental primary studies (if included). | Pages 7-17 |
|  | 22b | Provide a general interpretation of the results in the context of other evidence. | Pages 16-18 |
|  | 22c | Discuss any limitations of the evidence from systematic reviews, their primary studies, and supplemental primary studies (if included) included in the overview of reviews. Discuss any limitations of the overview of reviews methods used. | Pages 16-18 |
|  | 22d | Discuss implications for practice, policy, and future research (both systematic reviews and primary research). Consider the relevance of the findings to the end users of the overview of reviews, eg, healthcare providers, policymakers, patients, among others. | Pages 18 |
| **Other information** | | |  |
| Registration and protocol | 23a | Provide registration information for the overview of reviews, including register name and registration number, or state that the overview of reviews was not registered. | Page 4 |
|  | 23b | Indicate where the overview of reviews protocol can be accessed, or state that a protocol was not prepared. | Page 4 |
|  | 23c | Describe and explain any amendments to information provided at registration or in the protocol. Indicate the stage of the overview of reviews at which amendments were made. | N/A |
| Support | 24 | Describe sources of financial or non-financial support for the overview of reviews, and the role of the funders or sponsors in the overview of reviews. | Cover page |
| Competing interests | 25 | Declare any competing interests of the overview of reviews' authors. | Cover page |
| Author information | 26a | Provide contact information for the corresponding author. | Cover page |
|  | 26b | Describe the contributions of individual authors and identify the guarantor of the overview of reviews. | Cover page |
| Availability of data and other materials | 27 | Report which of the following are available, where they can be found, and under which conditions they may be accessed: template data collection forms; data collected from included systematic reviews and supplemental primary studies; analytic code; any other materials used in the overview of reviews. | Page 7 |

**Supplementary Table S3.** Search strategy

| DATABASE |  | SEARCH STRATEGY | RESULTS |
| --- | --- | --- | --- |
| PUBMED | #1 | (((((((((("100% juice"[Title/Abstract]) OR ("100% fruit juice"[Title/Abstract])) OR ("100% citrus juice"[Title/Abstract])) OR ("100% vegetable juice"[Title/Abstract])) OR (100% juice[Title/Abstract])) OR ("fresh vegetable juice"[Title/Abstract])) OR ("fresh fruit juice"[Title/Abstract])) OR ("fresh juice"[Title/Abstract])) OR ("vegetable juice"[Title/Abstract])) OR ("fruit juice"[Title/Abstract]) | 3,467 |
|  | #2 | (((((((("systematic literature review") OR ("systematic review")) OR ("systematic")) OR ("meta-analysis")) OR ("meta-analyses")) OR ("meta-analysis")) OR ("meta-analyses")) OR ("literature review")) OR ("scientific integrity review")) OR (“evidence based medicine”) | 818,830 |
|  | #3 | (((juice, fruit[MeSH Terms]) OR (juices, fruit[MeSH Terms])) OR (juice, vegetable[MeSH Terms])) OR (juices, vegetable[MeSH Terms]) | 2,683 |
|  | #4 | #1 OR #3 | 5,562 |
|  | #5 | #2 AND #4 | 132 |
| EMBASE | 1 | ("100% juice" or "100% fruit juice" or "100% citrus juice" or "100% vegetable juice" or 100% juice or "fresh vegetable juice" or "fresh fruit juice" or "fresh juice" or “pure juice” or vegetable juice or fruit juice).mp. [mp=title, abstract, heading word, drug trade name, original title, device manufacturer, drug manufacturer, device trade name, keyword heading word, floating subheading word, candidate term word] | 9,971 |
|  | 2 | ("systematic literature review" or "systematic review" or "systematic" or "meta-analysis" or "meta-analyses" or "meta-analysis" or "meta-analyses" or "literature review" or "scientific integrity review" or “evidence based medicine”).mp. [mp=title, abstract, heading word, drug trade name, original title, device manufacturer, drug manufacturer, device trade name, keyword heading word, floating subheading word, candidate term word] | 1,146,033 |
|  | 3 | 1 and 2 | 266 |
| CINAHL | S1 | TI “100% juice” OR AB “100% juice” OR TI “100% fruit juice” OR AB “100% fruit juice” OR TI “100% citrus juice” OR AB “100% citrus juice” OR TI “100% vegetable juice” OR AB “100% vegetable juice” | 217 |
|  | S2 | TI 100% juice OR AB 100% juice OR TI “fresh vegetable juice” OR AB “fresh vegetable juice” OR TI “fresh fruit juice” OR AB “fresh fruit juice” OR TI “pure juice” OR AB “pure juice” | 306 |
|  | S3 | TI fruit juice OR AB fruit juice OR TI vegetable juice OR AB vegetable juice | 1,577 |
|  | S4 | TI “systematic literature review” OR AB “systematic literature review” OR TI “systematic review” OR AB “systematic review” OR TI “systematic” OR AB “systematic” OR TI “meta-analysis” OR AB “meta-analysis” OR TI “meta-analyses” OR AB “meta-analyses” OR TI “meta-analysis” OR AB “meta-analysis” | 222,056 |
|  | S5 | TI “meta-analyses” OR AB “meta-analyses” OR TI “literature review” OR AB “literature review” OR TI “scientific integrity review” OR AB “scientific integrity review” OR TI “evidence based medicine” OR AB “evidence based medicine” | 70,506 |
|  | S6 | PT systematic review OR PT scientific integrity review OR PT meta-annalys* OR PT literature review | 134,984 |
|  | S7 | S1 OR S2 OR S3 | 1,663 |
|  | S8 | S4 OR S5 OR S6 | 297,994 |
|  | S9 | S7 AND S8 | 78 |
| The Cochrane Library | #1 | ("100% juice" OR "100% fruit juice" OR "100% citrus juice" OR "100% vegetable juice" OR 100% juice OR "fresh vegetable juice" OR "fresh fruit juice" OR "fresh juice" OR "vegetable juice" OR "fruit juice" OR “pure juice”):ti,ab,kw (Word variations have been searched) | 1,553 |
|  | #2 | MeSH descriptor: [Fruit and Vegetable Juices] explode all trees | 336 |
|  | #3 | ("systematic literature review" OR "systematic review" OR "systematic" OR "meta-analysis" OR "meta-analyses" OR "meta-analysis" OR "meta-analyses" OR "literature review" OR "scientific integrity review" OR “evidence based medicine”):ti,ab,kw | 38,516 |
|  | #4 | #1 OR #2 | 1,553 |
|  | #5 | #3 AND #4 | 22 |

**Supplementary Table S4.** Inter-rater reliability between reviewers at full text review.

| **Reviewer A** | **Reviewer B** | **A Include,**  **B Include** | **A Include,**  **B Exclude** | **A Exclude,**  **B Include** | **A Exclude,**  **B Exclude** | **Proportionate Agreement** | **Yes Probability** | **No Probability** | **Random Agreement Probability** | **Cohen’s Kappa** |
| --- | --- | --- | --- | --- | --- | --- | --- | --- | --- | --- |
| EB | MB | 15 | 8 | 4 | 54 | 0.85 | 0.07 | 0.55 | 0.61 | 0.62 |

**Supplementary Table S5.** Studies excluded after full-text review

| FIRST AUTHOR | TITLE | YEAR | JOURNAL | VOL (ISSUE) | PAGES | EXCLUSION REASON |
| --- | --- | --- | --- | --- | --- | --- |
| Zamani^1^ | The benefits and risks of beetroot juice consumption: a systematic review | 2021 | Crit Rev Food Sci Nutr | 61 (5) | 788-804 | Wrong Study Design |
| Yeary^2^ | Sweet Beverages and Cancer: A Scoping Review of Quantitative Studies | 2022 | Cancer Prevention Research | 15 (6) | 377-390 | Wrong Study Design |
| Xia^3^ | Consumption of cranberry as adjuvant therapy for urinary tract infections in susceptible populations: A systematic review and meta-analysis with trial sequential analysis | 2021 | PLoS ONE | 16 (9) | e0256992 | Not 100% Juice |
| Wang^4^ | The effects of pomegranate supplementation on biomarkers of inflammation and endothelial dysfunction: A meta-analysis and systematic review | 2020 | Complement Ther Med | 49 | 102358 | Not 100% Juice |
| Wang^5^ | Fruit and Vegetable Intake and Mortality Results From 2 Prospective Cohort Studies of US Men and Women and a Meta-Analysis of 26 Cohort Studies | 2021 | Circulation | 143 (17) | 1642-1654 | Not 100% Juice |
| Wang^6^ | Effect of fruit juice on glucose control and insulin sensitivity in adults: A meta-analysis of 12 randomized controlled trials | 2014 | PLoS ONE | 9 (4) | e95323 | Not 100% Juice |
| Virgen-Carrillo^7^ | Potential Hypoglycemic Effect of Pomegranate Juice and Its Mechanism of Action: A Systematic Review | 2020 | Journal of Medicinal Food | 23 (1) | 1-Nov | Wrong Study Design |
| Urbaniak^8^ | Effect of pomegranate fruit supplementation on performance and various markers in athletes and active subjects: A systematic review | 2021 | International Journal for Vitamin and Nutrition Research | 91 | 547-561 | Wrong Study Design |
| A. Khan T; Chiavaroli^9^ | A lack of consideration of a dose-response relationship can lead to erroneous conclusions regarding 100% fruit juice and the risk of cardiometabolic disease | 2019 | European Journal of Clinical Nutrition | 73 (12) | 1556-1560 | Wrong Study Design |
| Singh, G^10^ | Global, regional, and national consumption of sugar-sweetened beverages, fruit juices, and milk: A systematic assessment of beverage intake in 187 countries | 2015 | PLoS ONE | 10 (8) | e0124845 | Wrong Study Design |
| Silva^11^ | Factors that Moderate the Effect of Nitrate Ingestion on Exercise Performance in Adults: A Systematic Review with Meta-Analyses and Meta-Regressions | 2022 | Advances in Nutrition | 13 (5) | 1866-1881 | Wrong Study Design |
| Semnani-Azad^12^ | Association of Major Food Sources of Fructose-Containing Sugars with Incident Metabolic Syndrome: A Systematic Review and Meta-analysis | 2020 | JAMA Network Open | 3 (7) | e209993 | Wrong Study Design |
| Salas^13^ | Diet influenced tooth erosion prevalence in children and adolescents: Results of a meta-analysis and meta-regression | 2015 | Journal of Dentistry | 43 (8) | 865-875 | Not 100% Juice |
| Sakaki^14^ | Fruit juice and childhood obesity: a review of epidemiologic studies | 2022 | Critical reviews in food science and nutrition |  | Jan-15 | Wrong Study Design |
| Ruxton^15^ | Pure 100% fruit juices - more than just a source of free sugars? A review of the evidence of their effect on risk of cardiovascular disease, type 2 diabetes and obesity | 2021 | Nutrition Bulletin | 46 (4) | 415-431 | Wrong Study Design |
| Ruxton^16^ | Daily consumption of 100% orange juice does not increase body weight in adults: A meta-analysis of randomised controlled trials | 2020 | Proceedings of the Nutrition Society | 79 |  | Conference Abstract |
| Rojas-Valverde^17^ | Effectiveness of beetroot juice derived nitrates supplementation on fatigue resistance during repeated-sprints: a systematic review | 2021 | Critical reviews in food science and nutrition | 61 (20) | 3395-3406 | Wrong Study Design |
| Rickards^18^ | Effect of Polyphenol-Rich Foods, Juices, and Concentrates on Recovery from Exercise Induced Muscle Damage: A Systematic Review and Meta-Analysis | 2021 | Nutrients | 13 (9) |  | Wrong Study Design |
| Rampersaud^19^ | 100% citrus juice: Nutritional contribution, dietary benefits, and association with anthropometric measures | 2017 | Critical reviews in food science and nutrition | 57 (1) | 129-140 | Wrong Study Design |
| Murphy^20^ | 100% Fruit juice and measures of glycemic control and insulin sensitivity: A meta-analysis of randomized controlled trials | 2016 | FASEB Journal | 30 |  | Conference Abstract |
| Motallaei^21^ | Effects of orange juice intake on cardiovascular risk factors: A systematic review and meta-analysis of randomized controlled clinical trials | 2021 | Phytother Res | 35 (10) | 5427-5439 | Not 100% Juice |
| Mayer-Davis^22^ | Beverage Consumption and Growth, Size, Body Composition, and Risk of Overweight and Obesity: A Systematic Review. | 2020 | USDA Nutrition Evidence Systematic Reviews |  |  | Wrong Study Design |
| Mahboubi^23^ | Dietary free sugar and dental caries in children: A systematic review on longitudinal studies | 2021 | Health Promotion Perspectives | 11 (3) | 270-280 | Wrong Study Design |
| Liu^24^ | Effect of Fruit Juice on Cholesterol and Blood Pressure in Adults: A Meta-Analysis of 19 Randomized Controlled Trials | 2013 | PLoS ONE | 8 (4) | e61420 | Not 100% Juice |
| Liska^25^ | 100% Fruit Juice and Dental Health: A Systematic Review of the Literature | 2019 | Front Public Health | 7 | 190 | Wrong Study Design |
| Li^26^ | Consumption of sugar-sweetened beverages and fruit juice and human cancer: A systematic review and dose-response meta-analysis of observational studies | 2021 | Journal of Cancer | 12 (10) | 3077-3088 | Wrong Study Design |
| Li^27^ | Medical nutritional therapy for patients with gout and hyperuricemia: a systemic review | 2017 | Chinese Journal of Clinical Nutrition | 25 (1) | Dec-21 | Not In English |
| Li^28^ | Dietary Factors Associated with Dental Erosion: A Meta-Analysis | 2012 | PLoS ONE | 7 (8) | e42626 | Not 100% Juice |
| Lamport^29^ | Fruits, vegetables, 100% juices, and cognitive function | 2014 | Nutrition Reviews | 72 (12) | 774-789 | Wrong Study Design |
| Kazemi^30^ | The relationship between major food sources of fructose and cardiovascular disease, cancer, and all-cause mortality: a systematic review and dose-response meta-analysis of cohort studies | 2021 | Crit Rev Food Sci Nutr |  | 41640.00 | Not 100% Juice |
| Kalan Farmanfarma^31^ | Epidemiologic Study of Gastric Cancer in Iran: A Systematic Review | 2020 | Clin Exp Gastroenterol | 13 | 511-542 | Wrong Study Design |
| Imamura^32^ | Consumption of sugar sweetened beverages, artificially sweetened beverages, and fruit juice and incidence of type 2 diabetes: Systematic review, meta-analysis, and estimation of population attributable fraction | 2015 | BMJ (Online) | 351 | h3576 | Wrong Study Design |
| Hoon^33^ | The effect of nitrate supplementation on exercise performance in healthy individuals: A systematic review and meta-analysis | 2013 | International Journal of Sport Nutrition and Exercise Metabolism | 23 (5) | 522-532 | Not 100% Juice |
| Hoare^34^ | Sugar- and Intense-Sweetened Drinks in Australia: A Systematic Review on Cardiometabolic Risk | 2017 | Nutrients | 9 (10) | 1075 | Wrong Study Design |
| Hebden^35^ | Fruit consumption and adiposity status in adults: A systematic review of current evidence | 2017 | Critical reviews in food science and nutrition | 57 (12) | 2526-2540 | Wrong Study Design |
| Guzek^36^ | Fruit and Vegetable Dietary Patterns and Mental Health in Women: A Systematic Review | 2022 | Nutrition Reviews | 80 (6) | 1357-1370 | Wrong Study Design |
| English^36^ | Types and Amounts of Complementary Foods and Beverages and Growth, Size, and Body Composition: A Systematic Review | 2019 | American journal of clinical nutrition; USDA Nutrition Evidence Systematic Reviews |  |  | Wrong Study Design |
| Grassi^37^ | Pomegranate juice and blood pressure: A systematic review and meta-analysis of randomized controlled trials | 2016 | Journal of Hypertension | 34 (Supplement 2) | e109 | Conference Abstract |
| Gbinigie^38^ | Evidence for the effectiveness of pomegranate supplementation for blood pressure management is weak: A systematic review of randomized clinical trials | 2017 | Nutr Res | 46 | 38-48 | Wrong Study Design |
| Gao^39^ | Effect of Tart Cherry Concentrate on Endurance Exercise Performance: A Meta-analysis | 2020 | J Am Coll Nutr | 39 (7) | 657-664 | Not 100% Juice |
| Frantsve-Hawley^40^ | A systematic review of the association between consumption of sugar-containing beverages and excess weight gain among children under age 12 | 2017 | Journal of public health dentistry | 77 (Supplement 1) | S43-S66 | Wrong Study Design |
| Farvid^40^ | Fruit and vegetable consumption and incident breast cancer: a systematic review and meta-analysis of prospective studies | 2021 | British Journal of Cancer | 125 (2) | 284-298 | Not 100% Juice |
| Fardet^40^ | Association between consumption of fruit or processed fruit and chronic diseases and their risk factors: a systematic review of meta-analyses | 2019 | Nutr Rev | 77 (6) | 376-387 | Wrong Study Design |
| Ebrahimpour- koujan^41^ | Consumption of sugar-sweetened beverages and serum uric acid concentrations: a systematic review and meta-analysis | 2021 | Journal of Human Nutrition & Dietetics | 34 (2) | 305-313 | Wrong Study Design |
| Domi­nguez^42^ | Effects of Beetroot Juice Supplementation on Cardiorespiratory Endurance in Athletes. A Systematic Review | 2017 | Nutrients | 9 (1) |  | Wrong Study Design |
| Di Marco^43^ | Modifiable Lifestyle Factors in Dementia: A Systematic Review of Longitudinal Observational Cohort Studies | 2014 | Journal of Alzheimer's Disease | 42 (1) | 119-135 | Wrong Study Design |
| Crowe-White^44^ | Metabolic impact of 100% fruit juice consumption on antioxidant/oxidant status and lipid profiles of adults: An Evidence-Based review | 2017 | Critical reviews in food science and nutrition | 57 (1) | 152-162 | Wrong Study Design |
| Crowe-White^45^ | Impact of 100% Fruit Juice Consumption on Diet and Weight Status of Children: An Evidence-based Review | 2016 | Critical reviews in food science and nutrition | 56 (5) | 871-884 | Wrong Study Design |
| Collese^46^ | Role of fruits and vegetables in adolescent cardiovascular health: A systematic review | 2017 | Nutrition Reviews | 75 (5) | 339-349 | Wrong Study Design |
| Coe^47^ | Impact of polyphenol-rich sources on acute postprandial glycaemia: A systematic review | 2016 | Journal of Nutritional Science | 5 | e24 | Wrong Study Design |
| Cirmi^48^ | Anticancer potential of Citrus juices and their extracts: A systematic review of both preclinical and clinical studies | 2017 | Frontiers in Pharmacology | 8 | 420 | Wrong Study Design |
| Cicero^49^ | Nutraceuticals and Blood Pressure Control: Results from Clinical Trials and Meta-Analyses | 2015 | High Blood Press Cardiovasc Prev | 22 (3) | 203-13 | Wrong Study Design |
| Choo^50^ | Food sources of fructose-containing sugars and glycaemic control: Systematic review and meta-analysis of controlled intervention studies | 2018 | The BMJ | 363 | k4644 | Not 100% Juice |
| Chen^51^ | Prevalence and beverage-related risk factors of gastroesophageal reflux disease: An original study in Chinese college freshmen, a systemic review and meta-analysis | 2022 | Neurogastroenterology and Motility | 34 (5) | e14266 | Not 100% Juice |
| Carvalho^52^ | Blueberry intervention improves metabolic syndrome risk factors: systematic review and meta-analysis | 2021 | Nutr Res | 91 | 67-80 | Not 100% Juice |
| Boyle^53^ | Sugar and fructose consumption and the risk of cancer | 2015 | Journal of Clinical Oncology | 33 (15 SUPPL. 1) |  | Conference Abstract |
| Bonilla Ocampo^54^ | Dietary Nitrate from Beetroot Juice for Hypertension: A Systematic Review | 2018 | Biomolecules | 8 (4) |  | Wrong Study Design |
| Bahadoran^55^ | The Nitrate-Independent Blood Pressure-Lowering Effect of Beetroot Juice: A Systematic Review and Meta-Analysis | 2017 | Advances in nutrition (Bethesda, Md.) | 8 (6) | 830-838 | Inappropriate Comparator |
| Ayoub-Charette^56^ | Important food sources of fructose-containing sugars and incident gout: A systematic review and meta-analysis of prospective cohort studies | 2019 | BMJ Open | 9 (5) | e024171 | Not 100% Juice |
| Auerbach^57^ | Review of 100% Fruit Juice and Chronic Health Conditions: Implications for Sugar-Sweetened Beverage Policy | 2018 | Advances in nutrition (Bethesda, Md.) | 9 (2) | 78-85 | Wrong Study Design |
| Assis^58^ | Effects of fruit juice intake on inflammatory and oxidative stress related promoter genes | 2012 | Journal of Nutrigenetics and Nutrigenomics | 5 | 234 | Conference Abstract |
| Asadi-Pooya^59^ | Caffeinated drinks, fruit juices, and epilepsy: A systematic review | 2022 | Acta Neurol Scand | 145 (2) | 127-138 | Wrong Study Design |
| Amagase | Meta-analysis of the general effects of a standardized Lycium barbarum fruit juice shown in randomized, double-blind, placebocontrolled human clinical studies | 2009 | The FASEB Journal | 23 (S1) |  | Conference Abstract |

S1 Zamani, H. *et al.* The benefits and risks of beetroot juice consumption: a systematic review. *Crit Rev Food Sci Nutr* **61**, 788-804 (2021). https://doi.org:10.1080/10408398.2020.1746629

S2 Yeary, K. H. K. *et al.* Sweet Beverages and Cancer: A Scoping Review of Quantitative Studies. *Cancer Prev Res (Phila)* **15**, 377-390 (2022). https://doi.org:10.1158/1940-6207.Capr-21-0507

S3 Xia, J. Y. *et al.* Consumption of cranberry as adjuvant therapy for urinary tract infections in susceptible populations: A systematic review and meta-analysis with trial sequential analysis. *PLoS One* **16**, e0256992 (2021). https://doi.org:10.1371/journal.pone.0256992

S4 Wang, P. *et al.* The effects of pomegranate supplementation on biomarkers of inflammation and endothelial dysfunction: A meta-analysis and systematic review. *Complement Ther Med* **49**, 102358 (2020). https://doi.org:10.1016/j.ctim.2020.102358

S5 Wang, D. D. *et al.* Fruit and Vegetable Intake and Mortality: Results From 2 Prospective Cohort Studies of US Men and Women and a Meta-Analysis of 26 Cohort Studies. *Circulation* **143**, 1642-1654 (2021). https://doi.org:10.1161/circulationaha.120.048996

S6 Wang, B., Liu, K., Mi, M. & Wang, J. Effect of fruit juice on glucose control and insulin sensitivity in adults: a meta-analysis of 12 randomized controlled trials. *PLoS One* **9**, e95323 (2014). https://doi.org:10.1371/journal.pone.0095323

S7 Potential Hypoglycemic Effect of Pomegranate Juice and Its Mechanism of Action: A Systematic Review. *Journal of Medicinal Food* **23**, 1-11 (2020). https://doi.org:10.1089/jmf.2019.0069

S8 Urbaniak, A. & Skarpańska-Stejnborn, A. Effect of pomegranate fruit supplementation on performance and various markers in athletes and active subjects: A systematic review. *Int J Vitam Nutr Res* **91**, 547-561 (2021). https://doi.org:10.1024/0300-9831/a000601

S9 Khan, T. A., Chiavaroli, L., Zurbau, A. & Sievenpiper, J. L. A lack of consideration of a dose-response relationship can lead to erroneous conclusions regarding 100% fruit juice and the risk of cardiometabolic disease. *Eur J Clin Nutr* **73**, 1556-1560 (2019). https://doi.org:10.1038/s41430-019-0514-x

S10 Singh, G. M. *et al.* Global, Regional, and National Consumption of Sugar-Sweetened Beverages, Fruit Juices, and Milk: A Systematic Assessment of Beverage Intake in 187 Countries. *PLoS One* **10**, e0124845 (2015). https://doi.org:10.1371/journal.pone.0124845

S11 Silva, K. V. C., Costa, B. D., Gomes, A. C., Saunders, B. & Mota, J. F. Factors that Moderate the Effect of Nitrate Ingestion on Exercise Performance in Adults: A Systematic Review with Meta-Analyses and Meta-Regressions. *Adv Nutr* **13**, 1866-1881 (2022). https://doi.org:10.1093/advances/nmac054

S12 Semnani-Azad, Z. *et al.* Association of Major Food Sources of Fructose-Containing Sugars With Incident Metabolic Syndrome: A Systematic Review and Meta-analysis. *JAMA Netw Open* **3**, e209993 (2020). https://doi.org:10.1001/jamanetworkopen.2020.9993

S13 Salas, M. M. *et al.* Diet influenced tooth erosion prevalence in children and adolescents: Results of a meta-analysis and meta-regression. *J Dent* **43**, 865-875 (2015). https://doi.org:10.1016/j.jdent.2015.05.012

S14 Sakaki, J. R. *et al.* Fruit juice and childhood obesity: a review of epidemiologic studies. *Crit Rev Food Sci Nutr* **63**, 6723-6737 (2023). https://doi.org:10.1080/10408398.2022.2044284

S15 Ruxton, C. H. S., Derbyshire, E. & Sievenpiper, J. L. Pure 100% fruit juices – more than just a source of free sugars? A review of the evidence of their effect on risk of cardiovascular disease, type 2 diabetes and obesity. *Nutrition Bulletin* **46**, 415-431 (2021). https://doi.org:https://doi.org/10.1111/nbu.12526

S16 Ruxton, C., Horgan, G. & De Rycker, J. Daily consumption of 100% orange juice does not increase body weight in adults: a meta-analysis of randomised controlled trials. *Proceedings of the Nutrition Society* **79**, E227 (2020). https://doi.org:10.1017/S0029665120001755

S17 Rojas-Valverde, D., Montoya-Rodríguez, J., Azofeifa-Mora, C. & Sanchez-Urena, B. Effectiveness of beetroot juice derived nitrates supplementation on fatigue resistance during repeated-sprints: a systematic review. *Crit Rev Food Sci Nutr* **61**, 3395-3406 (2021). https://doi.org:10.1080/10408398.2020.1798351

S18 Rickards, L. *et al.* Effect of Polyphenol-Rich Foods, Juices, and Concentrates on Recovery from Exercise Induced Muscle Damage: A Systematic Review and Meta-Analysis. *Nutrients* **13** (2021). https://doi.org:10.3390/nu13092988

S19 Rampersaud, G. C. & Valim, M. F. 100% citrus juice: Nutritional contribution, dietary benefits, and association with anthropometric measures. *Crit Rev Food Sci Nutr* **57**, 129-140 (2017). https://doi.org:10.1080/10408398.2013.862611

S20 Murphy, M. M., Barrett, E. C., Bresnahan, K. A. & Barraj, L. M. 100 % Fruit juice and measures of glucose control and insulin sensitivity: a systematic review and meta-analysis of randomised controlled trials. *J Nutr Sci* **6**, e59 (2017). https://doi.org:10.1017/jns.2017.63

S21 Motallaei, M. *et al.* Effects of orange juice intake on cardiovascular risk factors: A systematic review and meta-analysis of randomized controlled clinical trials. *Phytother Res* **35**, 5427-5439 (2021). https://doi.org:10.1002/ptr.7173

S22 Mayer-Davis E, L. H., Mattes R, Naimi T, Novotny R, Schneeman B, Kingshipp BJ, Spill M, Cole NC, Bahnfleth CL, Butera G, Terry N, Obbagy J. . Beverage Consumption and Growth, Size, Body Composition, and Risk of Overweight and Obesity: A Systematic Review. . *U.S. Department of Agriculture, Food and Nutrition Service, Center for Nutrition Policy and Promotion, Nutrition Evidence Systematic Review. Available at: https://doi.org/10.52570/NESR.DGAC2020.SR0401* (2020).

S23 Mahboobi, Z., Pakdaman, A., Yazdani, R., Azadbakht, L. & Montazeri, A. Dietary free sugar and dental caries in children: A systematic review on longitudinal studies. *Health Promot Perspect* **11**, 271-280 (2021). https://doi.org:10.34172/hpp.2021.35

S24 Liu, K. *et al.* Effect of fruit juice on cholesterol and blood pressure in adults: a meta-analysis of 19 randomized controlled trials. *PLoS One* **8**, e61420 (2013). https://doi.org:10.1371/journal.pone.0061420

S25 Liska, D., Kelley, M. & Mah, E. 100% Fruit Juice and Dental Health: A Systematic Review of the Literature. *Front Public Health* **7**, 190 (2019). https://doi.org:10.3389/fpubh.2019.00190

S26 Li, Y., Guo, L., He, K., Huang, C. & Tang, S. Consumption of sugar-sweetened beverages and fruit juice and human cancer: a systematic review and dose-response meta-analysis of observational studies. *J Cancer* **12**, 3077-3088 (2021). https://doi.org:10.7150/jca.51322

S27 Li, R. & Yu, K. Medical nutritional therapy for patients with gout and hyperuricemia: a systemic review. *Chinese Journal of Clinical Nutrition* **25**, 12-21 (2017). https://doi.org:10.3760/cma.j.issn.1674-635X.2017.01.002

S28 Li, H., Zou, Y. & Ding, G. Dietary factors associated with dental erosion: a meta-analysis. *PLoS One* **7**, e42626 (2012). https://doi.org:10.1371/journal.pone.0042626

S29 Lamport, D. J., Saunders, C., Butler, L. T. & Spencer, J. P. Fruits, vegetables, 100% juices, and cognitive function. *Nutr Rev* **72**, 774-789 (2014). https://doi.org:10.1111/nure.12149

S30 Kazemi, A. *et al.* The relationship between major food sources of fructose and cardiovascular disease, cancer, and all-cause mortality: a systematic review and dose-response meta-analysis of cohort studies. *Crit Rev Food Sci Nutr* **63**, 4274-4287 (2023). https://doi.org:10.1080/10408398.2021.2000361

S31 Kalan Farmanfarma, K., Mahdavifar, N., Hassanipour, S. & Salehiniya, H. Epidemiologic Study of Gastric Cancer in Iran: A Systematic Review. *Clin Exp Gastroenterol* **13**, 511-542 (2020). https://doi.org:10.2147/ceg.S256627

S32 Imamura, F. *et al.* Consumption of sugar sweetened beverages, artificially sweetened beverages, and fruit juice and incidence of type 2 diabetes: systematic review, meta-analysis, and estimation of population attributable fraction. *Bmj* **351**, h3576 (2015). https://doi.org:10.1136/bmj.h3576

S33 Hoon, M. W., Johnson, N. A., Chapman, P. G. & Burke, L. M. The effect of nitrate supplementation on exercise performance in healthy individuals: a systematic review and meta-analysis. *Int J Sport Nutr Exerc Metab* **23**, 522-532 (2013). https://doi.org:10.1123/ijsnem.23.5.522

S34 Hoare, E. *et al.* Sugar- and Intense-Sweetened Drinks in Australia: A Systematic Review on Cardiometabolic Risk. *Nutrients* **9** (2017). https://doi.org:10.3390/nu9101075

S35 Hebden, L. *et al.* Fruit consumption and adiposity status in adults: A systematic review of current evidence. *Crit Rev Food Sci Nutr* **57**, 2526-2540 (2017). https://doi.org:10.1080/10408398.2015.1012290

S36 Guzek, D., Gła Bska, D., Groele, B. & Gutkowska, K. Fruit and Vegetable Dietary Patterns and Mental Health in Women: A Systematic Review. *Nutr Rev* **80**, 1357-1370 (2022). https://doi.org:10.1093/nutrit/nuab007

S37 Grassi, D., Sahebkar, A. & Ferri, S. [OP.8E.05] POMEGRANATE JUICE AND BLOOD PRESSURE: A SYSTEMATIC REVIEW AND META-ANALYSIS OF RANDOMIZED CONTROLLED TRIALS. *Journal of Hypertension* **34** (2016).

S38 Gbinigie, O. A., Onakpoya, I. J. & Spencer, E. A. Evidence for the effectiveness of pomegranate supplementation for blood pressure management is weak: A systematic review of randomized clinical trials. *Nutr Res* **46**, 38-48 (2017). https://doi.org:10.1016/j.nutres.2017.07.007

S39 Gao, R. & Chilibeck, P. D. Effect of Tart Cherry Concentrate on Endurance Exercise Performance: A Meta-analysis. *J Am Coll Nutr* **39**, 657-664 (2020). https://doi.org:10.1080/07315724.2020.1713246

S40 Frantsve-Hawley, J., Bader, J. D., Welsh, J. A. & Wright, J. T. A systematic review of the association between consumption of sugar-containing beverages and excess weight gain among children under age 12. *J Public Health Dent* **77 Suppl 1**, S43-s66 (2017). https://doi.org:10.1111/jphd.12222

S41 Ebrahimpour-Koujan, S., Saneei, P., Larijani, B. & Esmaillzadeh, A. Consumption of sugar-sweetened beverages and serum uric acid concentrations: a systematic review and meta-analysis. *J Hum Nutr Diet* **34**, 305-313 (2021). https://doi.org:10.1111/jhn.12796

S42 Domínguez, R. *et al.* Effects of Beetroot Juice Supplementation on Cardiorespiratory Endurance in Athletes. A Systematic Review. *Nutrients* **9** (2017). https://doi.org:10.3390/nu9010043

S43 Di Marco, L. Y. *et al.* Modifiable lifestyle factors in dementia: a systematic review of longitudinal observational cohort studies. *J Alzheimers Dis* **42**, 119-135 (2014). https://doi.org:10.3233/jad-132225

S44 Crowe-White, K. *et al.* Metabolic impact of 100% fruit juice consumption on antioxidant/oxidant status and lipid profiles of adults: An Evidence-Based review. *Crit Rev Food Sci Nutr* **57**, 152-162 (2017). https://doi.org:10.1080/10408398.2015.1102861

S45 Crowe-White, K. *et al.* Impact of 100% Fruit Juice Consumption on Diet and Weight Status of Children: An Evidence-based Review. *Crit Rev Food Sci Nutr* **56**, 871-884 (2016). https://doi.org:10.1080/10408398.2015.1061475

S46 Collese, T. S. *et al.* Role of fruits and vegetables in adolescent cardiovascular health: a systematic review. *Nutr Rev* **75**, 339-349 (2017). https://doi.org:10.1093/nutrit/nux002

S47 Coe, S. & Ryan, L. Impact of polyphenol-rich sources on acute postprandial glycaemia: a systematic review. *J Nutr Sci* **5**, e24 (2016). https://doi.org:10.1017/jns.2016.11

S48 Cirmi, S. *et al.* Anticancer Potential of Citrus Juices and Their Extracts: A Systematic Review of Both Preclinical and Clinical Studies. *Front Pharmacol* **8**, 420 (2017). https://doi.org:10.3389/fphar.2017.00420

S49 Cicero, A. F. & Colletti, A. Nutraceuticals and Blood Pressure Control: Results from Clinical Trials and Meta-Analyses. *High Blood Press Cardiovasc Prev* **22**, 203-213 (2015). https://doi.org:10.1007/s40292-015-0081-8

S50 Choo, V. L. *et al.* Food sources of fructose-containing sugars and glycaemic control: systematic review and meta-analysis of controlled intervention studies. *Bmj* **363**, k4644 (2018). https://doi.org:10.1136/bmj.k4644

S51 Chen, Y. *et al.* Prevalence and beverage-related risk factors of gastroesophageal reflux disease: An original study in Chinese college freshmen, a systemic review and meta-analysis. *Neurogastroenterol Motil* **34**, e14266 (2022). https://doi.org:10.1111/nmo.14266

S52 Carvalho, M. F., Lucca, A. B. A., Ribeiro, E. S. V. R., Macedo, L. R. & Silva, M. Blueberry intervention improves metabolic syndrome risk factors: systematic review and meta-analysis. *Nutr Res* **91**, 67-80 (2021). https://doi.org:10.1016/j.nutres.2021.04.006

S53 Boyle, P. *et al.* Sugar and fructose consumption and the risk of cancer. *Journal of Clinical Oncology* **33**, e12586-e12586 (2015). https://doi.org:10.1200/jco.2015.33.15_suppl.e12586

S54 Bonilla Ocampo, D. A. *et al.* Dietary Nitrate from Beetroot Juice for Hypertension: A Systematic Review. *Biomolecules* **8** (2018). https://doi.org:10.3390/biom8040134

S55 Bahadoran, Z., Mirmiran, P., Kabir, A., Azizi, F. & Ghasemi, A. The Nitrate-Independent Blood Pressure-Lowering Effect of Beetroot Juice: A Systematic Review and Meta-Analysis. *Adv Nutr* **8**, 830-838 (2017). https://doi.org:10.3945/an.117.016717

S56 Ayoub-Charette, S. *et al.* Important food sources of fructose-containing sugars and incident gout: a systematic review and meta-analysis of prospective cohort studies. *BMJ Open* **9**, e024171 (2019). https://doi.org:10.1136/bmjopen-2018-024171

S57 Auerbach, B. J., Dibey, S., Vallila-Buchman, P., Kratz, M. & Krieger, J. Review of 100% Fruit Juice and Chronic Health Conditions: Implications for Sugar-Sweetened Beverage Policy. *Adv Nutr* **9**, 78-85 (2018). https://doi.org:10.1093/advances/nmx006

S58 Assis. Effects of fruit juice intake on inflammatory and oxidative stress related promoter genes, https://karger.com/Article/PDF/343955. *J Nutrigenetics & Nutrigenomics* **5, 234** (2012).

S59 Asadi-Pooya, A. A., Zeraatpisheh, Z., Rostaminejad, M. & Damabi, N. M. Caffeinated drinks, fruit juices, and epilepsy: A systematic review. *Acta Neurol Scand* **145**, 127-138 (2022). https://doi.org:10.1111/ane.13544

**Supplementary Table S6.** SLRs excluded due to overlap.

| CITATION | OUTCOME | OVERLAP WITH |
| --- | --- | --- |
| Xi, B.; Li, S.; Liu, Z.; Tian, H.; Yin, X.; Huai, P.; Tang, W.; Zhou, D.; Steffen, L. M. Intake of fruit juice and incidence of type 2 diabetes: A systematic review and meta-analysis; PLoS ONE 2014;9(3):e93471^60^ | Metabolic Health | Immamura et al & Dinu et al. |
| Halvorsen, R. E.; Elvestad, M.; Molin, M.; Aune, D. Fruit and vegetable consumption and the risk of type 2 diabetes: A systematic review and dose-response meta-analysis of prospective studies BMJ Nutrition, Prevention and Health 2021;4(2):519-531^61^ | Metabolic Health | Immamura et al & Dinu et al. |
| Rousham, E. K.; Goudet, S.; Markey, O.; Griffiths, P.; Boxer, B.; Carroll, C.; Petherick, E. S.; Pradeilles, R.Unhealthy Food and Beverage Consumption in Children and Risk of Overweight and Obesity: A Systematic Review and Meta-Analysis; Advances in nutrition (Bethesda, Md.) 2022;13(5):1669-1696^62^ | Body Composition (Children) | Auerbach et al. |

S60 Xi, B. *et al.* Intake of fruit juice and incidence of type 2 diabetes: a systematic review and meta-analysis. *PLoS One* **9**, e93471 (2014). https://doi.org:10.1371/journal.pone.0093471

S61 Halvorsen, R. E., Elvestad, M., Molin, M. & Aune, D. Fruit and vegetable consumption and the risk of type 2 diabetes: a systematic review and dose-response meta-analysis of prospective studies. *BMJ Nutr Prev Health* **4**, 519-531 (2021). https://doi.org:10.1136/bmjnph-2020-000218

S62 Rousham, E. K. *et al.* Unhealthy Food and Beverage Consumption in Children and Risk of Overweight and Obesity: A Systematic Review and Meta-Analysis. *Adv Nutr* **13**, 1669-1696 (2022). https://doi.org:10.1093/advances/nmac032

**Supplementary Table S7**. ROBIS summary

|  | **Phase 2: Identification of concerns** | | | | **Phase 3** |
| --- | --- | --- | --- | --- | --- |
| **Author (year)** | Eligibility Criteria | Identification & Selection | Data collection & appraisal | Synthesis & Findings | Risk of bias judgement |
| Asgary (2021) ^63^ | Low | Unclear | Unclear | Unclear | High |
| Auerbach (2017) ^64^ | Low | Low | Low | Low | Low |
| Ayoub-Charette (2021) ^65^ | Low | Low | Low | Low | Low |
| Cara (2022) ^66^ | Low | Unclear | Low | Low | Unclear |
| D'Elia (2021) ^67^ | Unclear | Low | Unclear | Unclear | Unclear |
| Imamura (2016) ^32^ | Low | Low | Low | Low | Low |
| Lee (2022) ^68^ | Low | Low | Low | Low | Low |
| Llaha (2021) ^69^ | Unclear | Low | Low | Unclear | Unclear |
| Murphy (2017) ^20^ | Unclear | Low | Low | Low | Low |
| Pan (2021) ^70^ | Low | Low | Low | Low | Low |
| Liu (2019) ^71^ | Low | Low | Low | Low | Low |
| Qi (2022) ^72^ | Low | Low | Low | Low | Low |
| Sahebkar (2016) ^73^ | Low | High | High | Unclear | High |
| Sahebkar (2017) ^74^ | Unclear | High | High | Unclear | High |
| Zurbau (2020) ^75^ | Low | Low | Low | Low | Low |

S20 Murphy, M. M., Barrett, E. C., Bresnahan, K. A. & Barraj, L. M. 100 % Fruit juice and measures of glucose control and insulin sensitivity: a systematic review and meta-analysis of randomised controlled trials. *J Nutr Sci* **6**, e59 (2017). https://doi.org:10.1017/jns.2017.63

S32 Imamura, F. *et al.* Consumption of sugar sweetened beverages, artificially sweetened beverages, and fruit juice and incidence of type 2 diabetes: systematic review, meta-analysis, and estimation of population attributable fraction. *Bmj* **351**, h3576 (2015). https://doi.org:10.1136/bmj.h3576

S63 Asgary, S. *et al.* Effect of pomegranate juice on vascular adhesion factors: A systematic review and meta-analysis. *Phytomedicine* **80**, 153359 (2021). https://doi.org:10.1016/j.phymed.2020.153359

S64 Auerbach, B. J. *et al.* Fruit Juice and Change in BMI: A Meta-analysis. *Pediatrics* **139** (2017). https://doi.org:10.1542/peds.2016-2454

S65 Ayoub-Charette, S. *et al.* Different Food Sources of Fructose-Containing Sugars and Fasting Blood Uric Acid Levels: A Systematic Review and Meta-Analysis of Controlled Feeding Trials. *J Nutr* **151**, 2409-2421 (2021). https://doi.org:10.1093/jn/nxab144

S66 Cara, K. C., Beauchesne, A. R., Wallace, T. C. & Chung, M. Effects of 100% Orange Juice on Markers of Inflammation and Oxidation in Healthy and At-Risk Adult Populations: A Scoping Review, Systematic Review, and Meta-analysis. *Adv Nutr* **13**, 116-137 (2022). https://doi.org:10.1093/advances/nmab101

S67 D'Elia, L., Dinu, M., Sofi, F., Volpe, M. & Strazzullo, P. 100% Fruit juice intake and cardiovascular risk: a systematic review and meta-analysis of prospective and randomised controlled studies. *Eur J Nutr* **60**, 2449-2467 (2021). https://doi.org:10.1007/s00394-020-02426-7

S68 Lee, D. *et al.* Important Food Sources of Fructose-Containing Sugars and Non-Alcoholic Fatty Liver Disease: A Systematic Review and Meta-Analysis of Controlled Trials. *Nutrients* **14** (2022). https://doi.org:10.3390/nu14142846

S69 Llaha, F. *et al.* Consumption of Sweet Beverages and Cancer Risk. A Systematic Review and Meta-Analysis of Observational Studies. *Nutrients* **13** (2021). https://doi.org:10.3390/nu13020516

S70 Pan, B. *et al.* Association of soft drink and 100% fruit juice consumption with all-cause mortality, cardiovascular diseases mortality, and cancer mortality: A systematic review and dose-response meta-analysis of prospective cohort studies. *Crit Rev Food Sci Nutr* **62**, 8908-8919 (2022). https://doi.org:10.1080/10408398.2021.1937040

S71 Liu, Q. *et al.* Important Food Sources of Fructose-Containing Sugars and Incident Hypertension: A Systematic Review and Dose-Response Meta-Analysis of Prospective Cohort Studies. *J Am Heart Assoc* **8**, e010977 (2019). https://doi.org:10.1161/jaha.118.010977

S72 Qi, X. *et al.* Effect of Important Food Sources of Fructose-Containing Sugars on Inflammatory Biomarkers: A Systematic Review and Meta-Analysis of Controlled Feeding Trials. *Nutrients* **14** (2022). https://doi.org:10.3390/nu14193986

S73 Sahebkar, A., Gurban, C., Serban, A., Andrica, F. & Serban, M. C. Effects of supplementation with pomegranate juice on plasma C-reactive protein concentrations: A systematic review and meta-analysis of randomized controlled trials. *Phytomedicine* **23**, 1095-1102 (2016). https://doi.org:10.1016/j.phymed.2015.12.008

S74 Sahebkar, A. *et al.* Effects of pomegranate juice on blood pressure: A systematic review and meta-analysis of randomized controlled trials. *Pharmacol Res* **115**, 149-161 (2017). https://doi.org:10.1016/j.phrs.2016.11.018

S75 Zurbau, A. *et al.* Relation of Different Fruit and Vegetable Sources With Incident Cardiovascular Outcomes: A Systematic Review and Meta-Analysis of Prospective Cohort Studies. *J Am Heart Assoc* **9**, e017728 (2020). https://doi.org:10.1161/jaha.120.017728

**Supplementary Table S8**. GRADE

| AUTHOR | YEAR REVIEW PUBLISHED | OUTCOME TYPE | JUICE TYPE (INTERVENTION) | INTERVENTION DURATION | INTERVENTION DOSE | META-ANALYSED OUTCOME | GRADE | GRADE REPORTED IN STUDY | PRIMARY/  SECONDARY |
| --- | --- | --- | --- | --- | --- | --- | --- | --- | --- |
| Asgary^63^ | 2021 | Inflammation | 100% Pomegranate juice | 2-48 weeks | 50-240 mL | IL-6 | Very low | NR | Primary |
| Asgary^63^ | 2021 | CVD Risk Factors | 100% Pomegranate juice | 2-12 weeks | 150-250 mL | ICAM-1 | Low | NR | Primary |
| Asgary^63^ | 2021 | CVD risk factors | 100% Pomegranate juice | 2-12 weeks | 150-250 mL | E-Selectin | Very low | NR | Primary |
| Asgary^63^ | 2021 | CVD risk factors | 100% Pomegranate juice | 2-12 weeks | 150-250 mL | VCAM-1 | Very low | NR | Primary |
| Auerbach^64^ | 2017 | Body composition | 100% fruit juice | 2-6 years | 1 serve/day increment categories | BMI z score | Very low | NR | Primary |
| Auerbach^64^ | 2017 | Body composition | 100% fruit juice | 2-3 years | 1 serve/day increment categories | BMI z score | Very low | NR | Secondary |
| Auerbach^64^ | 2017 | Body composition | 100% fruit juice | 2-6 years | 1 serve/day increment categories | BMI z score | Very low | NR | Secondary |
| Ayoub-Charette^65^ | 2021 | CVD | 100% fruit juice | 1 - 12 weeks | 120mL - 1200mL/d | Uric acid levels (mg/dL) | Medium | High | Primary |
| Cara^66^ | 2022 | Inflammation | 100% Orange Juice | 5 hours - 4 weeks | 240-1000 mL/day | IL-6 | Very low | Very Low | Primary |
| Cara^66^ | 2022 | Inflammation | 100% Orange Juice | 1-12 weeks | 500-1000 mL/day | CRP | Very low | Very Low | Primary |
| Cara^66^ | 2022 | Inflammation | 100% Orange Juice | 2.5 hours - 12 weeks | 500 mL/day | MDA | Very low | Very Low | Primary |
| Cara^66^ | 2022 | Inflammation | 100% Orange Juice | 5-7 hours | 240-500 mL/day | IL-6 | Very low | Very Low | Secondary |
| Cara^66^ | 2022 | Inflammation | 100% Orange Juice | 1-4 weeks | 500-1000 mL/day | IL-6 | Very low | Very Low | Secondary |
| Cara^66^ | 2022 | Inflammation | 100% Orange Juice | 4-12 weeks | 500 mL/day | CRP | Very low | Very Low | Secondary |
| Cara^66^ | 2022 | Inflammation | 100% Orange Juice | 1-4 weeks | 500-1000 mL/day | CRP | Very low | Very Low | Secondary |
| Cara^66^ | 2022 | Inflammation | 100% Orange Juice | 4-12 weeks | 500-750 mL/day | MDA | Very low | Very Low | Secondary |
| D'Elia^67^ | 2021 | Blood Pressure | 100% Fruit Juice (various) | 1-16 weeks | 100-1000 mL/day | DBP | Low | High | Primary |
| D'Elia^67^ | 2021 | Blood Pressure | 100% Fruit Juice (various) | 1-16 weeks | 100-1000 mL/day | SBP | Medium | High | Primary |
| D'Elia^67^ | 2021 | Blood Pressure | 100% Pomegranate Juice | 1-13 weeks | 240-500 mL/day | DBP | Very low | NR | Secondary |
| D'Elia^67^ | 2021 | Blood Pressure | 100% Pomegranate Juice | 1-13 weeks | 240-500 mL/day | SBP | Very low | NR | Secondary |
| D'Elia^67^ | 2021 | Blood Pressure | 100% Blackcurrant Juice | 6 weeks | 1000 mL/day | DBP | Very low | NR | Secondary |
| D'Elia^67^ | 2021 | Blood Pressure | 100% Blackcurrant Juice | 6 weeks | 1000 mL/day | SBP | Very low | NR | Secondary |
| D'Elia^67^ | 2021 | Blood Pressure | 100% Cherry Juice | 6-12 weeks | 250-480 mL/day | DBP | Very low | NR | Secondary |
| D'Elia^67^ | 2021 | Blood Pressure | 100% Cherry Juice | 6-12 weeks | 250-480 mL/day | SBP | Very low | NR | Secondary |
| D'Elia^67^ | 2021 | Blood Pressure | 100% Cranberry Juice | 4-16 weeks | 460-480 ml/day | SBP | Very low | NR | Secondary |
| D'Elia^67^ | 2021 | Blood Pressure | 100% Grape Juice | 2-16 weeks | 5.5--7.8 ml/kg/day; 100-150 mL | DBP | Very low | NR | Secondary |
| D'Elia^67^ | 2021 | Blood Pressure | 100% Grape Juice | 2-16 weeks | 5.5-7.8 ml/kg/day; 100-150 mL | SBP | Very low | NR | Secondary |
| D'Elia^67^ | 2021 | Blood Pressure | 100% Cranberry Juice | 4-16 weeks | 460-480 ml/day | DBP | Very low | NR | Secondary |
| D'Elia^67^ | 2021 | Body composition | 100% Fruit Juice (various) | 1-16 weeks | 120-1000 mL/day | Body Weight | Very low | NR | Primary |
| D'Elia^67^ | 2021 | Body composition | 100% Fruit Juice (various) | 4-12 weeks | 120-1000 ml/day | BMI | Very low | NR | Primary |
| D'Elia^67^ | 2021 | Body composition | 100% Fruit Juice (various) | 4-16 weeks | 120-500 mL/day | Waist Circumference | Very low | NR | Primary |
| D'Elia^67^ | 2021 | Body composition | 100% Blackcurrant Juice | 6 weeks | 1000 mL/day | BMI | Very low | NR | Secondary |
| D'Elia^67^ | 2021 | Body composition | 100% Blackcurrant Juice | 6 weeks | 1000 mL/day | Body Weight | Very low | NR | Secondary |
| D'Elia^67^ | 2021 | Body composition | 100% Pomegranate juice | 4-13 weeks | 120-250 mL/day | Body Weight | Very low | NR | Secondary |
| D'Elia^67^ | 2021 | Body composition | 100% Pomegranate juice | 4-6 weeks | 120-300 mL/day | Waist Circumference | Very low | NR | Secondary |
| D'Elia^67^ | 2021 | Body composition | 100% Pomegranate juice | 4-12 weeks | 120-330 mL/day | BMI | Very low | NR | Secondary |
| D'Elia^67^ | 2021 | Body composition | 100% Orange Juice | 1-12 weeks | 250-500 mL/day | Body Weight | Very low | NR | Secondary |
| D'Elia^67^ | 2021 | Body composition | 100% Grape Juice | 2-12 weeks | 5.5-7.8 mL/kg/day-480 mL/day | Body Weight | Very low | NR | Secondary |
| D'Elia^67^ | 2021 | Body composition | 100% Grape Juice | 8-12 weeks | 5.5 mL/kg/day-480 mL/day | BMI | Very low | NR | Secondary |
| D'Elia^67^ | 2021 | Body composition | 100% Grape Juice | 12-16 weeks | 6.3-7.8 mL/kg/day-480 mL/day | Waist Circumference | Very low | NR | Secondary |
| D'Elia^67^ | 2021 | Body composition | 100% Mixed juice | 6-8 weeks | 120-750 mL/d | Body Weight | Very low | NR | Secondary |
| D'Elia^67^ | 2021 | CVD | 100% fruit juice | 1 - 6 weeks | 7 mL/kg/d; 480 – 1000 mL/d | Flow-mediated dilation (%) | Medium | NR | Primary |
| D'Elia^67^ | 2021 | CVD | 100% fruit juice | 2 - 8 weeks | 7 mL/kg/d; 250 – 500 mL/d | Pulse wave velocity (m/s) | Very low | NR | Primary |
| D'Elia^67^ | 2021 | CVD Risk Factors | 100% fruit juice | 1 - 16 weeks | 120 – 1000 mL/d | Total cholesterol (mg/dl) | Low | NR | Primary |
| D'Elia^67^ | 2021 | CVD Risk Factors | 100% fruit juice | 1 - 13 weeks | 120 – 750 mL/d | LDL-cholesterol (mg/dl) | Low | NR | Primary |
| D'Elia^67^ | 2021 | CVD Risk Factors | 100% fruit juice | 1 - 16 weeks | 120 – 750 mL/d | Triglycerides (mg/dl) | Low | NR | Primary |
| D'Elia^67^ | 2021 | CVD Risk Factors | 100% fruit juice | 1 - 16 weeks | 120 – 750 mL/d | HDL-cholesterol (mg/dl) | Low | NR | Primary |
| D'Elia^67^ | 2021 | CVD Risk Factors | 100% blackcurrant juice | 6 weeks | 1000 mL/d | Total cholesterol (mg/dl) | Very low | NR | Secondary |
| D'Elia^67^ | 2021 | CVD Risk Factors | 100% pomegranate juice | 1 - 13 weeks | 120 – 500 mL/day | HDL-cholesterol (mg/dl) | Very low | NR | Secondary |
| D'Elia^67^ | 2021 | CVD Risk Factors | 100% pomegranate juice | 1 - 13 weeks | 120 – 500 mL/day | Triglycerides (mg/dl) | Very low | NR | Secondary |
| D'Elia^67^ | 2021 | CVD Risk Factors | 100% pomegranate juice | 1 - 13 weeks | 120 – 500 mL/day | LDL-cholesterol (mg/dl) | Very low | NR | Secondary |
| D'Elia^67^ | 2021 | CVD Risk Factors | 100% pomegranate juice | 1 - 13 weeks | 120 – 500 mL/day | Total cholesterol (mg/dl) | Very low | NR | Secondary |
| D'Elia^67^ | 2021 | CVD Risk Factors | 100% cherry juice | 6 - 12 weeks | 250 – 480 mL/d | HDL-cholesterol (mg/dl) | Very low | NR | Secondary |
| D'Elia^67^ | 2021 | CVD Risk Factors | 100% cherry juice | 6 - 12 weeks | 250 – 480 mL/d | Total cholesterol (mg/dl) | Very low | NR | Secondary |
| D'Elia^67^ | 2021 | CVD Risk Factors | 100% orange juice | 4 - 12 weeks | 250 – 500 mL/d | Triglycerides (mg/dl) | Very low | NR | Secondary |
| D'Elia^67^ | 2021 | CVD Risk Factors | 100% orange juice | 4 - 12 weeks | 250 – 500 mL/d | Total cholesterol (mg/dl) | Very low | NR | Secondary |
| D'Elia^67^ | 2021 | CVD Risk Factors | 100% orange juice | 4 -12 weeks | 250 – 500 mL/d | HDL-cholesterol (mg/dl) | Very low | NR | Secondary |
| D'Elia^67^ | 2021 | CVD Risk Factors | 100% orange juice | 4 -12 weeks | 250 – 500 mL/d | LDL-cholesterol (mg/dl) | Very low | NR | Secondary |
| D'Elia^67^ | 2021 | CVD Risk Factors | 100% cranberry juice | 2 - 16 weeks | 460 – 750 mL/day | Triglycerides (mg/dl) | Very low | NR | Secondary |
| D'Elia^67^ | 2021 | CVD Risk Factors | 100% cranberry juice | 2 - 16 weeks | 460 – 750 mL/day | Total cholesterol (mg/dl) | Very low | NR | Secondary |
| D'Elia^67^ | 2021 | CVD Risk Factors | 100% cranberry juice | 2 - 8 weeks | 480 – 750 mL/day | HDL-cholesterol (mg/dl) | Very low | NR | Secondary |
| D'Elia^67^ | 2021 | CVD Risk Factors | 100% cranberry juice | 2 - 8 weeks | 480 – 750 mL/day | LDL-cholesterol (mg/dl) | Very low | NR | Secondary |
| D'Elia^67^ | 2021 | CVD Risk Factors | 100% grape juice | 2 - 12 weeks | 5.5-7 ml/kg/d – 480 mL/day | Triglycerides (mg/dl) | Very low | NR | Secondary |
| D'Elia^67^ | 2021 | CVD Risk Factors | 100% grape juice | 2 - 12 weeks | 5.5-7 ml/kg/d – 480 mL/day | LDL-cholesterol (mg/dl) | Very low | NR | Secondary |
| D'Elia^67^ | 2021 | CVD Risk Factors | 100% grape juice | 2 - 12 weeks | 5.5-7 ml/kg/d – 480 mL/day | Total cholesterol (mg/dl) | Very low | NR | Secondary |
| D'Elia^67^ | 2021 | CVD Risk Factors | 100% grape juice | 8 - 12 weeks | 5.5-7mL/kg/d – 480 mL/day | HDL-cholesterol (mg/dl) | Very low | NR | Secondary |
| D'Elia^67^ | 2021 | Diabetes | 100% fruit juice | 1 - 12 weeks | 121 – 500 mL/d | HOMA Index (U) | Low | NR | Primary |
| D'Elia^67^ | 2021 | Diabetes | 100% fruit juice | 1 - 12 weeks | 121 – 500 mL/d | Insulin (%) | Low | NR | Primary |
| D'Elia^67^ | 2021 | Diabetes | 100% fruit juice | 8 - 13 weeks | 240 – 480 mL/d | Hba1c (%) | Low | NR | Primary |
| D'Elia^67^ | 2021 | Diabetes | 100% fruit juice | 1 - 16 weeks | 120 – 500 mL/d | Glucose (mg/dl) | Very low | NR | Primary |
| D'Elia^67^ | 2021 | Diabetes | 100% pomegranate juice | 1 - 13 weeks | 120 – 500 mL/d | Glucose (mg/dl) | Very low | NR | Secondary |
| D'Elia^67^ | 2021 | Diabetes | 100% cranberry juice | 4 - 12 weeks | 240 – 480 mL/d | Glucose (mg/dl) | Very low | NR | Secondary |
| D'Elia^67^ | 2021 | Diabetes | 100% pomegranate juice | 1 - 12 weeks | 250 – 500 mL/d | HOMA Index (U) | Very low | NR | Secondary |
| D'Elia^67^ | 2021 | Diabetes | 100% pomegranate juice | 1 - 12 weeks | 250 – 500 mL/d | Insulin (%) | Very low | NR | Secondary |
| D'Elia^67^ | 2021 | Diabetes | 100% orange juice | 4 -12 weeks | 250 – 500 mL/d | Glucose (mg/dl) | Very low | NR | Secondary |
| D'Elia^67^ | 2021 | Diabetes | 100% cranberry juice | 4 - 8 weeks | 480 mL/d | HOMA Index (U) | Very low | NR | Secondary |
| D'Elia^67^ | 2021 | Diabetes | 100% cranberry juice | 4 - 8 weeks | 480 mL/d | Insulin (%) | Very low | NR | Secondary |
| D'Elia^67^ | 2021 | Diabetes | 100% grape juice | 2 - 16 weeks | 5.5-7 ml/kg/d – 480 mL/d | Glucose (mg/dl) | Very low | NR | Secondary |
| D'Elia^67^ | 2021 | Diabetes | 100% grape juice | 8 weeks | 7mL/kg/d | Insulin (%) | Very low | NR | Secondary |
| Imamura^32^ | 2016 | Diabetes | 100% fruit juice | 5-21 years | Higher (serves/day) | Type 2 Diabetes Incidence | Very low | NR | Primary |
| Imamura^32^ | 2016 | Diabetes | 100% fruit juice | 5-21 years | Higher (serves/day) | Type 2 Diabetes Incidence | Very low | NR | Primary |
| Lee^68^ | 2022 | NAFLD | 100% fruit juice | 28 days - 6 weeks | 120-500 mL/day | ALT in addition trials (U/L) | Low | Medium | Primary |
| Lee^68^ | 2022 | NAFLD | 100% fruit juice | 28 days - 6 weeks | 120-500 mL/day | AST in addition trials (U/L) | Low | Medium | Primary |
| Lee^68^ | 2022 | NAFLD | 100% orange juice | 12 weeks | 500 mL/day | ALT in substitution trials (U/L) | Low | NR | Primary |
| Lee^68^ | 2022 | NAFLD | 100% orange juice | 12 weeks | 500 mL/day | AST in substitution trials (U/L) | Medium | NR | Primary |
| Llaha^69^ | 2021 | Cancer | 100% fruit juice | 4-15 years | Highest | Prostate cancer | Very low | NR | Primary |
| Llaha^69^ | 2021 | Cancer | 100% fruit juice | 4-6 years | Highest | Breast cancer | Very low | NR | Primary |
| Llaha^69^ | 2021 | Cancer | 100% fruit juice | 4-7 years | Highest | Colorectal cancer | Very low | NR | Primary |
| Murphy^20^ | 2017 | Diabetes | 100% fruit juice | 4 weeks - 6 months | 120-595 mL/d | Fasting blood insulin | Low | NR | Primary |
| Murphy^20^ | 2017 | Diabetes | 100% fruit juice | 4 weeks - 3 months | 150-250 mL/d | HbA1c | Low | NR | Primary |
| Murphy^20^ | 2017 | Diabetes | 100% fruit juice | 4 weeks - 6 months | 120-595 mL/d | Fasting blood glucose | Very low | NR | Primary |
| Murphy^20^ | 2017 | Diabetes | 100% fruit juice | 4 weeks - 6 months | 250-595 mL/d | HOMA-IR | Very low | NR | Primary |
| Murphy^20^ | 2017 | Diabetes | 100% pomegranate juice | 4 - 12 weeks | 250-500 mL/d | HOMA-IR | Low | NR | Secondary |
| Murphy | 2017 | Diabetes | 100% fruit juice | 2-7 weeks | 120-500 mL/d | Fasting blood glucose | Very low | NR | Secondary |
| Murphy | 2017 | Diabetes | 100% fruit juice | 2-7 weeks | 500 mL/d | HOMA-IR | Very low | NR | Secondary |
| Murphy^20^ | 2017 | Diabetes | 100% fruit juice | 4 weeks - 6 months | > 250 mL/d | Fasting blood insulin | Low | NR | Secondary |
| Murphy^20^ | 2017 | Diabetes | 100% fruit juice | 2-7 weeks | 120-500 mL/d | Fasting blood insulin | Low | NR | Secondary |
| Murphy^20^ | 2017 | Diabetes | 100% pomegranate juice | 4-12 weeks | 120-500 mL/d | Fasting blood glucose | Low | NR | Secondary |
| Murphy^20^ | 2017 | Diabetes | 100% grape juice | 4-16 weeks | 150-595 mL/d | Fasting blood insulin | Low | NR | Secondary |
| Murphy^20^ | 2017 | Diabetes | 100% fruit juice | >= 8 weeks | 240-595 mL/d | Fasting blood insulin | Low | NR | Secondary |
| Murphy^20^ | 2017 | Diabetes | 100% pomegranate juice | 3 months | 250-500 mL/d | HbA1c | Low | NR | Secondary |
| Murphy^20^ | 2017 | Diabetes | 100% citrus juice | 4 weeks - 6 months | 250-500 mL/d | Fasting blood insulin | Low | NR | Secondary |
| Murphy^20^ | 2017 | Diabetes | 100% fruit juice | >= 8 weeks | 250-595 mL/d | HOMA-IR | Low | NR | Secondary |
| Murphy^20^ | 2017 | Diabetes | 100% fruit juice | 12 weeks | <= 250 mL/d | HOMA-IR | Very low | NR | Secondary |
| Murphy^20^ | 2017 | Diabetes | 100% fruit juice | 4-12 weeks | <= 250 mL/d | Fasting blood glucose | Very low | NR | Secondary |
| Murphy^20^ | 2017 | Diabetes | 100% fruit juice | 4-12 weeks | <= 250 mL/d | Fasting blood insulin | Very low | NR | Secondary |
| Murphy^20^ | 2017 | Diabetes | 100% fruit juice | 4 weeks - 6 months | > 250 mL/d | Fasting blood glucose | Very low | NR | Secondary |
| Murphy^20^ | 2017 | Diabetes | 100% fruit juice | 4 weeks - 6 months | > 250 mL/d | HOMA-IR | Very low | NR | Secondary |
| Murphy^20^ | 2017 | Diabetes | 100% pomegranate juice | 4-12 weeks | 120-500 mL/d | Fasting blood insulin | Very low | NR | Secondary |
| Murphy^20^ | 2017 | Diabetes | 100% grape juice | 4-16 weeks | 150-595 mL/d | Fasting blood glucose | Very low | NR | Secondary |
| Murphy^20^ | 2017 | Diabetes | 100% fruit juice | >= 8 weeks | 240-250 mL/d | HbA1c | Very low | NR | Secondary |
| Murphy^20^ | 2017 | Diabetes | 100% berry juice | 4-12 weeks | 240-500 mL/d | Fasting blood glucose | Very low | NR | Secondary |
| Murphy^20^ | 2017 | Diabetes | 100% fruit juice | >= 8 weeks | 240-595 mL/d | Fasting blood glucose | Very low | NR | Secondary |
| Murphy^20^ | 2017 | Diabetes | 100% citrus juice | 4 weeks - 6 months | 250-500 mL/d | HOMA-IR | Very low | NR | Secondary |
| Murphy^20^ | 2017 | Diabetes | 100% citrus juice | 4 weeks -6 months | 250-500 mL/d | Fasting blood glucose | Very low | NR | Secondary |
| Pan^70^ | 2021 | CVD | 100% fruit juice | 16 years | Highest | CVD Mortality | Very low | Very low | Primary |
| Pan^70^ | 2021 | CVD | 100% fruit juice | 11 years | Highest | All-Cause Mortality | Very low | Very low | Primary |
| Pan^70^ | 2021 | CVD | 100% fruit juice | 11 years | Dose Response: 250 mL/day | All-Cause Mortality | Very low | Very low | Secondary |
| Liu^71^ | 2019 | Blood Pressure | 100% Fruit Juice (various) | 7-20 years | Highest | Hypertension | Very low | Low | Primary |
| Qi^72^ | 2022 | Inflammation | 100% Fruit Juice (various) - substitution trials | 2 weeks | 500 mL/day | CRP | Medium | Low | Primary |
| Qi^72^ | 2022 | Inflammation | 100% Fruit Juice (various) - addition trials | 2-13 weeks | 100-700 mL/day | CRP | Low | Moderate | Primary |
| Qi^72^ | 2022 | Inflammation | 100% Fruit Juice (various) - addition trials | 2-13 weeks | 150-700 mL/day | IL-6 | Very low | Moderate | Primary |
| Qi^72^ | 2022 | Inflammation | 100% Fruit Juice (various) - addition trials | 4-9 weeks | 200g – 700 mL/day | TNF-alpha | Very low | Low | Primary |
| Sahebkar^74^ | 2017 | Blood Pressure | 100% Pomegranate juice | 2 weeks - 18 months | 50-500 mL/day | DBP | Low | NR | Primary |
| Sahebkar^74^ | 2017 | Blood Pressure | 100% Pomegranate juice | 2 weeks - 18 months | 50-500 mL/day | SBP | Low | NR | Primary |
| Sahebkar^74^ | 2017 | Blood Pressure | 100% Pomegranate juice | <12 weeks | 150-500 mL/day | SBP | Low | NR | Secondary |
| Sahebkar^74^ | 2017 | Blood Pressure | 100% Pomegranate juice | >12 weeks | 50-240 mL/day | SBP | Low | NR | Secondary |
| Sahebkar^74^ | 2017 | Blood Pressure | 100% Pomegranate juice | 2 weeks - 1 year | <240 mL/day | SBP | Medium | NR | Secondary |
| Sahebkar^74^ | 2017 | Blood Pressure | 100% Pomegranate juice | 4 weeks - 18 months | >240 mL/day | SBP | Very low | NR | Secondary |
| Sahebkar^74^ | 2017 | Blood Pressure | 100% Pomegranate juice | <12 weeks | 150-500 mL/day | DBP | Very low | NR | Secondary |
| Sahebkar^74^ | 2017 | Blood Pressure | 100% Pomegranate juice | 2 weeks - 1 year | <240 mL/day | DBP | Very low | NR | Secondary |
| Sahebkar^74^ | 2017 | Blood Pressure | 100% Pomegranate juice | 4 weeks - 18 months | >240 mL/day | DBP | Very low | NR | Secondary |
| Sahebkar^74^ | 2017 | Blood Pressure | 100% Pomegranate juice | >12 weeks | 50-240 mL/day | DBP | Very low | NR | Secondary |
| Sahebkar^74^ | 2016 | Inflammation | 100% Pomegranate juice | 2 weeks - 18 months | 150-250 mL/day | CRP | Very low | NR | Primary |
| Zurbau^75^ | 2020 | CVD | 100% fruit juice | 16 years | Highest | Stroke Mortality | Very low | Low | Primary |
| Zurbau^75^ | 2020 | CVD | 100% fruit juice | 7-19 years | Highest | CHD Incidence | Low | Very low | Primary |
| Zurbau^75^ | 2020 | CVD | 100% fruit juice | 8-16 years | Highest | CVD Incidence | Low | Very low | Primary |
| Zurbau^75^ | 2020 | CVD | 100% fruit juice | 6-17 years | Highest | CHD Mortality | Very low | Very low | Primary |
| Zurbau^75^ | 2020 | CVD | 100% fruit juice | 8-16 years | Highest | Stroke Incidence | Very low | Very low | Primary |
| Zurbau^75^ | 2020 | CVD | 100% fruit juice | 7-19 years | Dose Response servings/day | CHD Incidence | Low | Very low | Secondary |
| Zurbau^75^ | 2020 | CVD | 100% fruit juice | 7-19 years | Dose Response servings/day | CVD Incidence | Low | Very low | Secondary |
| Zurbau^75^ | 2020 | CVD | 100% fruit juice | 16 years | Dose Response servings/day | Stroke Mortality | Very low | Low | Secondary |
| Zurbau^75^ | 2020 | CVD | 100% fruit juice | 6-17 years | Dose Response servings/day | CHD Mortality | Very low | Very low | Secondary |
| Zurbau^75^ | 2020 | CVD | 100% fruit juice | 8-16 years | Dose Response servings/day | Stroke Incidence | Very low | Very low | Secondary |

S20 Murphy, M. M., Barrett, E. C., Bresnahan, K. A. & Barraj, L. M. 100 % Fruit juice and measures of glucose control and insulin sensitivity: a systematic review and meta-analysis of randomised controlled trials. *J Nutr Sci* **6**, e59 (2017). https://doi.org:10.1017/jns.2017.63

S32 Imamura, F. *et al.* Consumption of sugar sweetened beverages, artificially sweetened beverages, and fruit juice and incidence of type 2 diabetes: systematic review, meta-analysis, and estimation of population attributable fraction. *Bmj* **351**, h3576 (2015). https://doi.org:10.1136/bmj.h3576

S63 Asgary, S. *et al.* Effect of pomegranate juice on vascular adhesion factors: A systematic review and meta-analysis. *Phytomedicine* **80**, 153359 (2021). https://doi.org:10.1016/j.phymed.2020.153359

S64 Auerbach, B. J. *et al.* Fruit Juice and Change in BMI: A Meta-analysis. *Pediatrics* **139** (2017). https://doi.org:10.1542/peds.2016-2454

S65 Ayoub-Charette, S. *et al.* Different Food Sources of Fructose-Containing Sugars and Fasting Blood Uric Acid Levels: A Systematic Review and Meta-Analysis of Controlled Feeding Trials. *J Nutr* **151**, 2409-2421 (2021). https://doi.org:10.1093/jn/nxab144

S66 Cara, K. C., Beauchesne, A. R., Wallace, T. C. & Chung, M. Effects of 100% Orange Juice on Markers of Inflammation and Oxidation in Healthy and At-Risk Adult Populations: A Scoping Review, Systematic Review, and Meta-analysis. *Adv Nutr* **13**, 116-137 (2022). https://doi.org:10.1093/advances/nmab101

S67 D'Elia, L., Dinu, M., Sofi, F., Volpe, M. & Strazzullo, P. 100% Fruit juice intake and cardiovascular risk: a systematic review and meta-analysis of prospective and randomised controlled studies. *Eur J Nutr* **60**, 2449-2467 (2021). https://doi.org:10.1007/s00394-020-02426-7

S68 Lee, D. *et al.* Important Food Sources of Fructose-Containing Sugars and Non-Alcoholic Fatty Liver Disease: A Systematic Review and Meta-Analysis of Controlled Trials. *Nutrients* **14** (2022). https://doi.org:10.3390/nu14142846

S69 Llaha, F. *et al.* Consumption of Sweet Beverages and Cancer Risk. A Systematic Review and Meta-Analysis of Observational Studies. *Nutrients* **13** (2021). https://doi.org:10.3390/nu13020516

S70 Pan, B. *et al.* Association of soft drink and 100% fruit juice consumption with all-cause mortality, cardiovascular diseases mortality, and cancer mortality: A systematic review and dose-response meta-analysis of prospective cohort studies. *Crit Rev Food Sci Nutr* **62**, 8908-8919 (2022). https://doi.org:10.1080/10408398.2021.1937040

S71 Liu, Q. *et al.* Important Food Sources of Fructose-Containing Sugars and Incident Hypertension: A Systematic Review and Dose-Response Meta-Analysis of Prospective Cohort Studies. *J Am Heart Assoc* **8**, e010977 (2019). https://doi.org:10.1161/jaha.118.010977

S72 Qi, X. *et al.* Effect of Important Food Sources of Fructose-Containing Sugars on Inflammatory Biomarkers: A Systematic Review and Meta-Analysis of Controlled Feeding Trials. *Nutrients* **14** (2022). https://doi.org:10.3390/nu14193986

S74 Sahebkar, A. *et al.* Effects of pomegranate juice on blood pressure: A systematic review and meta-analysis of randomized controlled trials. *Pharmacol Res* **115**, 149-161 (2017). https://doi.org:10.1016/j.phrs.2016.11.018

S75 Zurbau, A. *et al.* Relation of Different Fruit and Vegetable Sources With Incident Cardiovascular Outcomes: A Systematic Review and Meta-Analysis of Prospective Cohort Studies. *J Am Heart Assoc* **9**, e017728 (2020). https://doi.org:10.1161/jaha.120.017728

**Supplementary Table S9** - Results from primary MAs of 100% fruit juice interventions for Metabolic Health

| Outcome | 100% Juice type | Intervention time | Intervention dose (mL/day) | N interventions | N subjects | Graphic | Mean difference (intervention vs. control) | Lower 95% CI | Upper 95% CI | I^2^ | Reference |
| --- | --- | --- | --- | --- | --- | --- | --- | --- | --- | --- | --- |
| HOMA Index (U) | 100% fruit juice | 1 - 12 weeks | 121 - 500 | 11 | 487 |  | 0.01 | -0.28 | 0.3 | 0 | D’Elia^67^ |
| Insulin (%) | 100% fruit juice | 1 - 12 weeks | 121 - 500 | 11 | 1691 |  | 3.4 | -7.2 | 14 | 0 | D’Elia^67^ |
| Hba1c (%) | 100% fruit juice | 8 - 13 weeks | 240 - 480 | 3 | 120 |  | -0.1 | -0.31 | 0.1 | 0 | D’Elia^67^ |
| Glucose (mg/dl) | 100% fruit juice | 1 - 16 weeks | 120 - 500 | 23 | 845 |  | -1.01 | -4.02 | 2 | 69 | D’Elia^67^ |
| Fasting blood insulin | 100% fruit juice | 4 weeks - 6 months | 120-595 | 11 | 565 |  | -0.24 | -3.54 | 3.05 | 0 | Murphy^20^ |
| HbA1c | 100% fruit juice | 4 weeks - 3 months | 150-250 | 3 | 110 |  | -0.001 | -0.38 | 0.38 | 22.3 | Murphy^20^ |
| Fasting blood glucose | 100% fruit juice | 4 weeks - 6 months | 120-595 | 16 | 878 |  | -0.13 | -0.28 | 0.01 | 70.6 | Murphy^20^ |
| HOMA-IR | 100% fruit juice | 4 weeks - 6 months | 250-595 | 7 | 445 |  | -0.22 | -0.5 | 0.06 | 73.9 | Murphy^20^ |

S20 Murphy, M. M., Barrett, E. C., Bresnahan, K. A. & Barraj, L. M. 100 % Fruit juice and measures of glucose control and insulin sensitivity: a systematic review and meta-analysis of randomised controlled trials. *J Nutr Sci* **6**, e59 (2017). https://doi.org:10.1017/jns.2017.63

S67 D'Elia, L., Dinu, M., Sofi, F., Volpe, M. & Strazzullo, P. 100% Fruit juice intake and cardiovascular risk: a systematic review and meta-analysis of prospective and randomised controlled studies. *Eur J Nutr* **60**, 2449-2467 (2021). https://doi.org:10.1007/s00394-020-02426-7

**Supplemental Table S10** – Results from primary MAs of 100% fruit juice interventions for Body composition

| Outcome | 100% Juice type | Intervention dose (ml/day) | Intervention time | N interventions | N subjects | Graphic | Mean difference (intervention vs. control) | Lower 95% CI | Upper 95% CI | I^2^ | Reference |
| --- | --- | --- | --- | --- | --- | --- | --- | --- | --- | --- | --- |
| Body Weight (kg) | 100% Fruit Juice (various) | 120-1000 | 1-16 weeks | 20 | 975 |  | -0.07 | -0.39 | 0.25 | 0 | D’Elia^67^ |
| BMI (kg/m2) | 100% Fruit Juice (various) | 120-1000 | 4-12 weeks | 13 | 588 |  | -0.03 | -0.15 | 0.1 | 0 | D’Elia^67^ |
| Waist Circumference (cm) | 100% Fruit Juice (various) | 120-500 | 4-16 weeks | 10 | 411 |  | 0.11 | -0.94 | 1.16 | 0 | D’Elia^67^ |

S67 D'Elia, L., Dinu, M., Sofi, F., Volpe, M. & Strazzullo, P. 100% Fruit juice intake and cardiovascular risk: a systematic review and meta-analysis of prospective and randomised controlled studies. *Eur J Nutr* **60**, 2449-2467 (2021). https://doi.org:10.1007/s00394-020-02426-7

**Supplemental Table S11.** Results from primary MAs of 100% fruit juice interventions for Liver Function

| Outcome | 100% juice type | Intervention dose (ml/day) | Intervention time | N interventions | N subjects | Graphic | Mean difference (intervention vs. Control) | Lower 95% ci | Upper 95% ci | I^2^ | Reference |
| --- | --- | --- | --- | --- | --- | --- | --- | --- | --- | --- | --- |
| ALT in addition trials (U/L) | 100% fruit juice | 120-500mL | 28 days - 6 weeks | 7 | 88 |  | -0.8 | -2.43 | 0.84 | 0 | Lee^68^ |
| AST in addition trials (U/L) | 100% fruit juice | 120-500mL | 28 days - 6 weeks | 3 | 65 |  | 0.02 | -2.66 | 2.71 | 0 | Lee^68^ |
| ALT in substitution trials (U/L) | 100% orange juice | 500mL per day | 12 weeks | 2 | 117 |  | -0.81 | -5.67 | 4.05 | 58.9 | Lee^68^ |
| AST in substitution trials (U/L) | 100% orange juice | 500mL per day | 12 weeks | 2 | 117 |  | -0.19 | -2.65 | 2.28 | 0 | Lee^68^ |

S68 Lee, D. *et al.* Important Food Sources of Fructose-Containing Sugars and Non-Alcoholic Fatty Liver Disease: A Systematic Review and Meta-Analysis of Controlled Trials. *Nutrients* **14** (2022). https://doi.org:10.3390/nu14142846
